# Single-molecule live-cell RNA imaging with CRISPR–Csm

**DOI:** 10.1038/s41587-024-02540-5

**Published:** 2025-02-18

**Authors:** Chenglong Xia, David Colognori, Xueyang Stephen Jiang, Ke Xu, Jennifer A. Doudna

**Affiliations:** 1 California Institute for Quantitative Biosciences (QB3), University of California, Berkeley, CA USA; 2 Innovative Genomics Institute, University of California, Berkeley, CA USA; 3 Department of Molecular and Cell Biology, University of California, Berkeley, CA USA; 4 Department of Chemistry, University of California, Berkeley, CA USA; 5 Howard Hughes Medical Institute, University of California, Berkeley, CA USA

**Keywords:** Single-molecule biophysics, Biochemistry

## Abstract

Understanding the diverse dynamic behaviors of individual RNA molecules in single cells requires visualizing them at high resolution in real time. However, single-molecule live-cell imaging of unmodified endogenous RNA has not yet been achieved in a generalizable manner. Here, we present single-molecule live-cell fluorescence in situ hybridization (smLiveFISH), a robust approach that combines the programmable RNA-guided, RNA-targeting CRISPR–Csm complex with multiplexed guide RNAs for direct and efficient visualization of single RNA molecules in a range of cell types, including primary cells. Using smLiveFISH, we track individual native *NOTCH2* and *MAP1B* transcripts in living cells and identify two distinct localization mechanisms including the cotranslational translocation of *NOTCH2* mRNA at the endoplasmic reticulum and directional transport of *MAP1B* mRNA toward the cell periphery. This method has the potential to unlock principles governing the spatiotemporal organization of native transcripts in health and disease.

## Abstract (web summary)

Single-molecule live-cell fluorescence in situ hybridization uses an RNA-targeting CRISPR–Csm complex to image and track endogenous RNAs.

## Main

RNA is directly involved in protein synthesis and regulates gene expression at both transcriptional and post-transcriptional levels^1^. Beyond RNA sequence, the spatial and temporal distributions of individual transcripts control these activities. Indeed, dynamic and orchestrated interactions among RNA, RNA-binding proteins (RBPs) and other cellular machinery occur at particular subcellular regions and time points^2–4^. For example, zipcode-binding protein 1 mediates directional transport of β-actin (ACTB) mRNA to the leading edge of fibroblasts^5^, where it becomes anchored to actin filaments by elongation factor 1α and locally translated, ultimately supporting cell growth and motility^6^.

Live-cell RNA imaging methods have begun to reveal RNA dynamics within individual cells, highlighting the value of such interrogations^2–4^. However, methods to label with stem loops^7,8^ or aptamers^9^ require the genetic insertion of sequences within specific regions of RNA or rely on exogenous expression of tagged RNA^7–9^—manipulations that are time-consuming and can interfere with native RNA behaviors^10^. Approaches to visualize unmodified endogenous RNA using molecular beacons^11^ or clustered regularly interspaced short palindromic repeats (CRISPR)–Cas systems^12–15^ suffer from limited single-molecule resolution (often restricted to highly abundant, repetitive RNAs)^3,4^ and excessive background signals produced by endosome-entrapped probes or overexpressed fluorescent protein fusions^3,4^. While the former methods have proven successful on occasion^16^, there is a pressing need for a generalizable single-molecule live-cell native RNA imaging platform.

Here, we describe single-molecule live-cell fluorescence in situ hybridization (smLiveFISH), an alternative strategy for visualizing any unmodified endogenous transcript. Using the RNA-targeting type III-A CRISPR–Csm system from *Streptococcus thermophilus* with multiplexed guide RNAs, smLiveFISH can track individual mRNA molecules in different types of living cells. We used smLiveFISH to analyze the behavior of two different mRNAs, *NOTCH2* and *MAP1B*, encoding a cell-surface receptor protein and a microtubule-associated protein, respectively. We found that *NOTCH2* mRNAs comprise two populations with distinct dynamics associated with cotranslational polypeptide translocation across the endoplasmic reticulum (ER). In contrast, *MAP1B* mRNAs exhibit several distinct behaviors including directional transport in a translation-independent manner. We further show that smLiveFISH can detect differences in single-transcript localization in response to small molecules, such as incorporation into P-bodies, underscoring the utility of assessing individual endogenous RNA behavior.

## Results

### Design and characterization of smLiveFISH

CRISPR–Cas complexes are programmable DNA or RNA nucleases from prokaryotic adaptive defense systems against bacteriophages^17,18^. Cas nucleases bind to CRISPR RNAs (crRNAs) to form ribonucleoprotein (RNP) complexes that recognize nucleic acid targets using base-pairing complementarity between the crRNA and target DNA or RNA^17,18^. Fluorescently labeled, catalytically inactivated Cas nucleases from different types of CRISPR–Cas systems (such as RCas9 (ref. ^12^), dCas13 (refs. ^13,14^) and dCsm (ref. ^15^)) have been used to label RNAs of interest in live cells. However, these approaches have yet to achieve single-molecule resolution because each RNA is targeted with a single crRNA, resulting in a similar fluorescence intensity between free and target-bound RNPs. Only RNA granules or RNAs with repeated sequences produce sufficient signal from multiple copies of bound RNPs that is distinguishable from nonspecific binding or background signal.

**Fig. 1:**
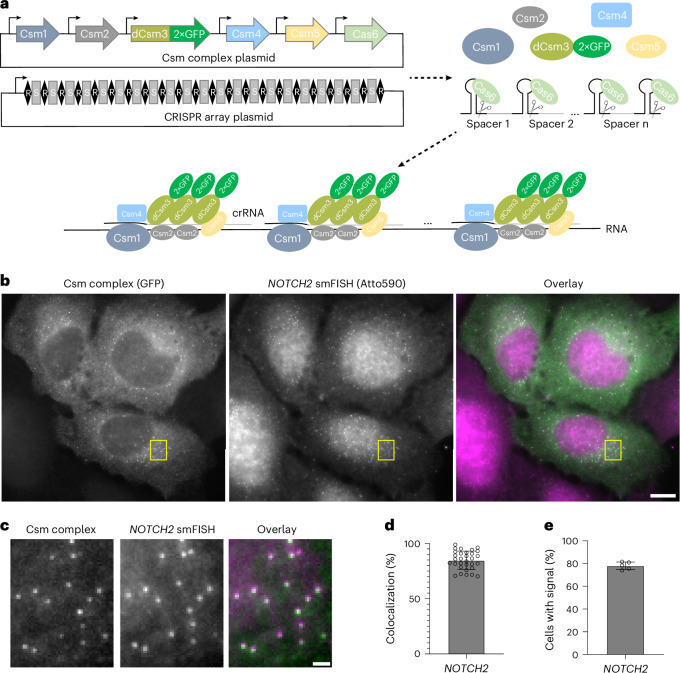
Imaging native single mRNA molecules with smLiveFISH. **a**, Schematic of the smLiveFISH system using multiplexed guides against a target RNA to achieve single-molecule resolution. Upon transfection with Csm and CRISPR array plasmids, cells produce Csm1, Csm2, dCsm3–2×GFP, Csm4, Csm5 and Cas6 proteins along with the pre-crRNA. Cas6 processes the pre-crRNA into individual crRNAs that assemble with Csm proteins into RNPs. RNPs, each with their own crRNA spacer, bind target RNA molecules simultaneously through base-pair complementarity, allowing RNA detection at single-molecule resolution. **b**, Left, fixed-cell image of individual *NOTCH2* mRNAs labeled by GFP-tagged Csm complex and 48 *NOTCH2*-targeting crRNAs. Middle, image of individual *NOTCH2* mRNAs labeled by smFISH probes. Right, overlaid image. Scale bar, 10 μm. **c**, Enlarged view of the yellow boxed region in **b**. Scale bar, 1 μm. **d**, Percentage colocalization of Csm complex foci and smFISH foci (measured as Csm complex foci colocalized with smFISH foci divided by Csm complex foci per cell). Error bar indicates the mean ± s.d.; each dot represents one cell; (*n* = 31 cells). **e**, Percentage of transfected cells with Csm-complex-labeled foci. Images obtained from five randomly selected 7 × 7 tiling regions from three biological replicates (*n* = 275 cells). Error bar indicates the mean ± s.d.; each dot represents one 7 × 7 tiling region.

To overcome this signal-to-noise issue, we drew inspiration from smFISH^19,20^. In smFISH^19,20^, short fluorescently labeled DNA probes are tiled along the target RNA, increasing the signal-to-noise ratio and allowing detection by microscopy of equal-intensity diffraction-limited spots in fixed cells^19,20^. We reasoned that single-molecule imaging of endogenous RNAs in living cells could be possible if fluorescently tagged RNPs can be simultaneously tiled along a target RNA (Fig. 1a).

**Extended Data Fig. 1:**
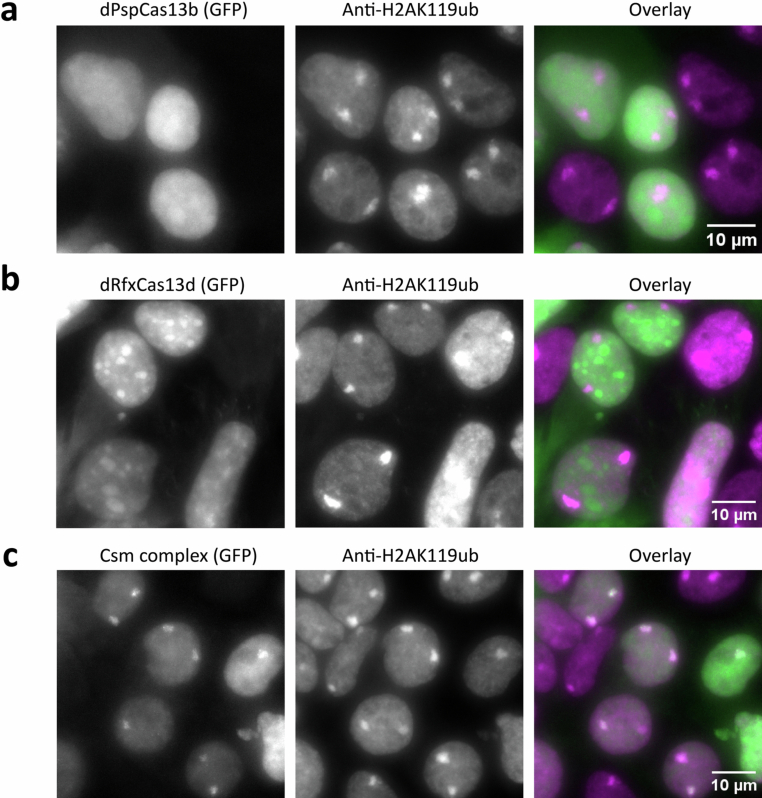
Testing different Cas proteins for RNA imaging. Representative images of GFP-tagged PspCas13b (**a**), RfxCas13d (**b**) and Csm complex (**c**) targeting *XIST* RNA in HEK293T cells (left), immunostaining for the heterochromatin marker H2AK119ub (middle), and their overlay (right). For each system, a single crRNA targeting the same repetitive sequence in *XIST* was used. Scale bar, 10 μm.

For initial studies we compared the GFP-fused Cas13 and Csm complexes, both of which can generate individual crRNAs by processing pre-crRNAs, as catalyzed by Cas13 (refs. ^21,22^) and Cas6 (ref. ^23^), respectively. To evaluate the labeling performance of dPspCas13b (ref. ^14^), dRfxCas13d (ref. ^22^) and dCsm (ref. ^15^) proteins, we targeted *XIST* RNA, a long noncoding transcript that forms large clouds from hundreds of *XIST* copies in HEK293T cell nuclei^24^. We used a single crRNA with eight complementary target sites on a repetitive region of *XIST*^15^ (Extended Data Fig. 1 and Supplementary Table 1) and validated labeling with H2AK119ub, a heterochromatin mark enriched at the inactivated X chromosome that overlaps with *XIST* RNA. Only the Csm complex could label *XIST* robustly, an observation consistent with prior results^15,25^ (Extended Data Fig. 1). Notably, the Csm system has other advantages relative to Cas13. First, it contains multiple GFP-linked catalytically inactive Csm3 molecules^15,26^ (≥3 per complex) (Fig. 1a), which enhances signal and may aid in single-molecule detection. Second, it has higher binding affinity for RNA (*K*_d_ = 0.3 nM)^23^ relative to that for Cas13 (*K*_d_ ≈ 10 nM)^27^. On the basis of these observations, we focused on the Csm complex to develop a single-molecule mRNA labeling system.

**Extended Data Fig. 2:**
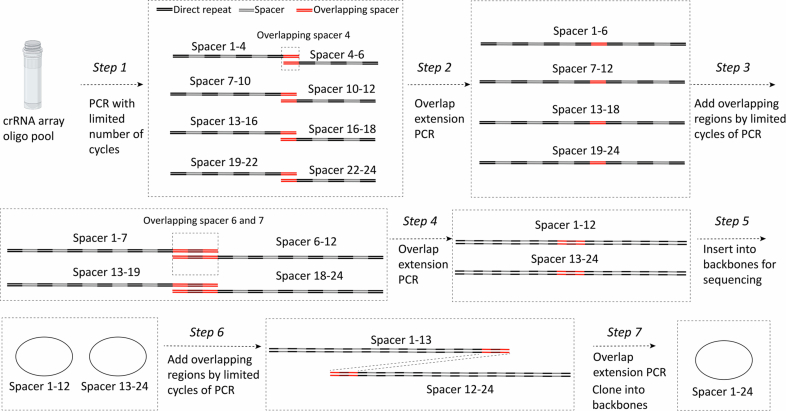
CRISPR array cloning strategy. Schematic of CRISPR array construction. First, each fragment from an oligo pool containing multiple 3-4 spacer fragments is amplified by limited-cycle PCR. Second, 6-spacer fragments are generated from the 3-4-spacer fragments by overlap extension PCR. Third, overhangs are added to the 6-spacer fragments. Fourth, 12-spacer fragments are generated from the 6-spacer fragments by overlap extension PCR. Fifth, the 12-spacer fragments are cloned into vectors for sequence verification. Sixth, overhangs are added to the 12-spacer fragments. Seventh, 24-spacer fragments are generated from the 12-spacer fragments by overlap extension PCR. Finally, the 24-spacer fragments are cloned into vectors for sequence verification. Black segments indicate CRISPR direct repeats; grey segments indicate spacers; red segments indicate the overlapping regions.

To target mRNAs in the cytoplasm, we removed the nuclear localization signal (NLS) from each protein component of the mammalian-optimized Csm system^15^ and again encoded them within a single plasmid (Fig. 1a and Supplementary Table 2). To facilitate the export of pre-crRNA from the nucleus to the cytoplasm, we added a short signal sequence^28^ present in naturally intronless mRNAs to the 5′ end of the CRISPR array and expressed it from a CAG (RNA polymerase II) promoter (Supplementary Table 2). We selected *NOTCH2* mRNA as a cytoplasmic target for proof of concept for two reasons. First, its length permits the design of distinct smFISH probes to validate labeling by the Csm complex. Second, it encodes a cell membrane protein and is, thus, enriched near the ER^29–31^, which is useful for RNA-centric exploration of cotranslational protein translocation. In each of two CRISPR array plasmids developed for this experiment (Extended Data Fig. 2 and Supplementary Table 2), all 24 36-nt targeting sequences were designed to bind in a tiled fashion along the length of the 3′ untranslated region (UTR) of *NOTCH2* mRNA to avoid potential interference with mRNA translation^32^.

**Extended Data Fig. 3:**
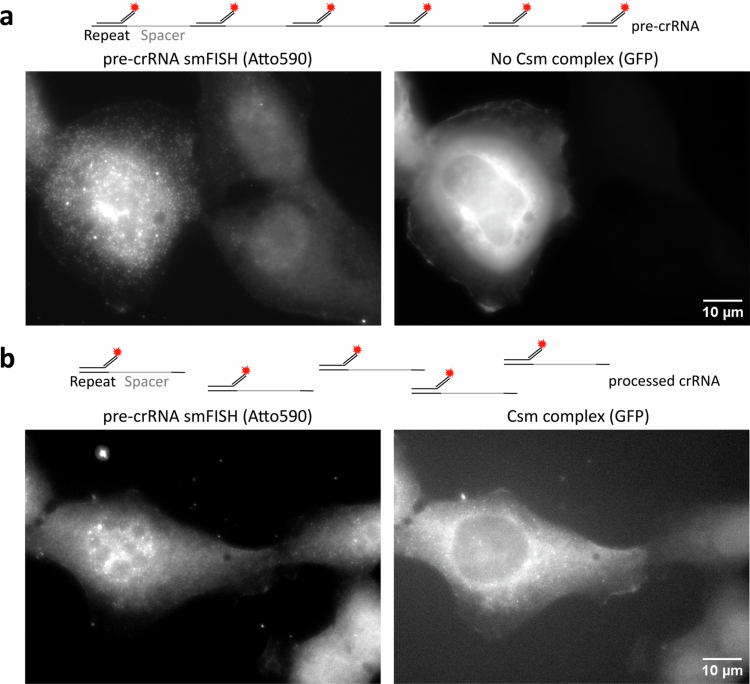
Verifying pre-cRNA processing in cells. **a**, Representative image of smFISH for pre-crRNA transcripts in U2OS cells co-transfected with plasmids encoding CRISPR array and GFP alone. Left, smFISH using a single FISH probe (Atto590) complementary to pre-crRNA direct repeats. Right, GFP channel for the same cells in left panel. **b**, Same as **a**, but cells were co-transfected with plasmids encoding CRISPR array and GFP-tagged Csm complex. Scale bar, 10 μm.

While CRISPR arrays were previously used for multiplexed targeting in human cells, their length was relatively short (generally fewer than ten crRNAs) and their processing was not directly demonstrated^15,33^. To test whether the Csm-associated Cas6 endonuclease could process long pre-crRNAs in cells (Fig. 1a), we used a single FISH probe (Extended Data Fig. 3a and Supplementary Table 3) complementary to the direct repeats to detect individual unprocessed pre-crRNAs. In U2OS cells cotransfected with a CRISPR-array-encoding plasmid and an empty vector expressing GFP alone, we observed high levels of intact pre-crRNA transcripts, indicated by diffraction-limited spots, in both the cytoplasm and the nucleus (Extended Data Fig. 3a). Upon cotransfection of plasmids encoding the CRISPR array and GFP-tagged Csm components, the individual spots disappeared specifically from the cytoplasm (where Cas6 and Csm proteins are localized), consistent with pre-crRNA processing (Extended Data Fig. 3b). Additionally, single-molecule spots were observed in the cytoplasm using GFP fluorescence detection, representing putative *NOTCH2* mRNA signals (Extended Data Fig. 3b).

### SmLiveFISH enables visualization of individual *NOTCH2* mRNAs

**Extended Data Fig. 4:**
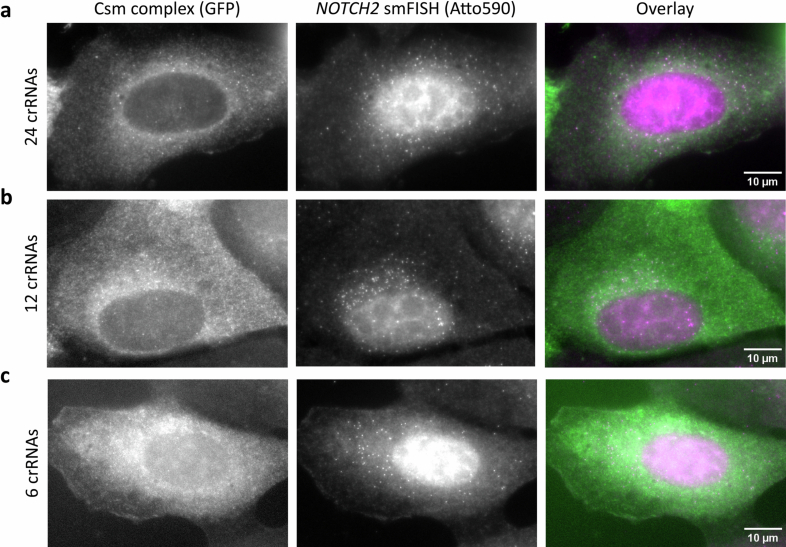
Varying the number of crRNAs for RNA imaging. *NOTCH2* mRNAs labeled by GFP-tagged Csm complex (left), smFISH (Atto590) (middle), and their overlay (right) using 24 (**a**), 12 (**b**), or 6 spacer-containing CRISPR arrays (**c**). Scale bar, 10 μm.

We next used *NOTCH2* smFISH to identify the spots observed in cells transfected with crRNA-array-encoding and Csm complex-encoding plasmids. Two-color imaging revealed strong colocalization of Csm-labeled foci with smFISH spots, indicating that GFP-tagged Csm complexes successfully labeled endogenous *NOTCH2* mRNA (Fig. 1b,c). Quantification showed that 85% of Csm-labeled spots colocalized with smFISH spots (Fig. 1d). In addition, we found that 78% of transfected cells had clearly distinguishable spots in the cytoplasm, consistent with a high labeling efficiency (Fig. 1e). We also tested whether fewer crRNAs (6, 12 or 24) could be used to efficiently label *NOTCH2* mRNA. Although we were able to detect single mRNA molecules using as few as six crRNAs (Extended Data Fig. 4a–c), the signal was less distinguishable from background compared to when more were used.

**Extended Data Fig. 5:**
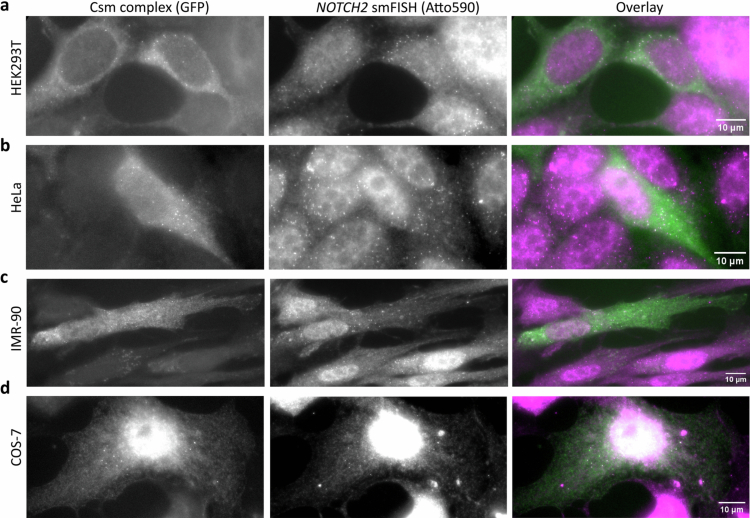
smLiveFISH works across various cell types. *NOTCH2* mRNAs labeled by GFP-tagged Csm complex (left), smFISH (Atto590) (middle), and their overlay (right) in HEK293T (**a**), HeLa (**b**), IMR-90 (**c**), and COS-7 cells (**d**). Scale bar, 10 μm.

**Extended Data Fig. 6:**
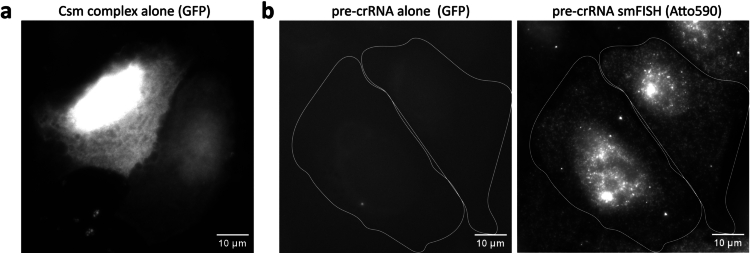
GFP signal is specific to mRNA labeling. Representative GFP signal in cells expressing GFP-tagged Csm complex alone (**a**) or pre-crRNA alone (**b**, left). Right panel of **b** shows smFISH (Atto590) for pre-crRNA transcripts. Scale bar, 10 μm.

We next applied this method in other cell lines, including HEK293T, HeLa, primary human fibroblast IMR-90 and African green monkey COS-7—the latter having 94% *NOTCH2* 3′ UTR sequence identity to the human sequence. We observed robust labeling of endogenous *NOTCH2* mRNA in all of these cell types (Extended Data Fig. 5a–d). As controls, expression of the Csm complex alone only generated a homogeneous signal when GFP fluorescence was monitored (Extended Data Fig. 6a) and expression of the crRNA array alone exhibited only weak homogeneous GFP autofluorescence (Extended Data Fig. 6b).

**Extended Data Fig. 7:**
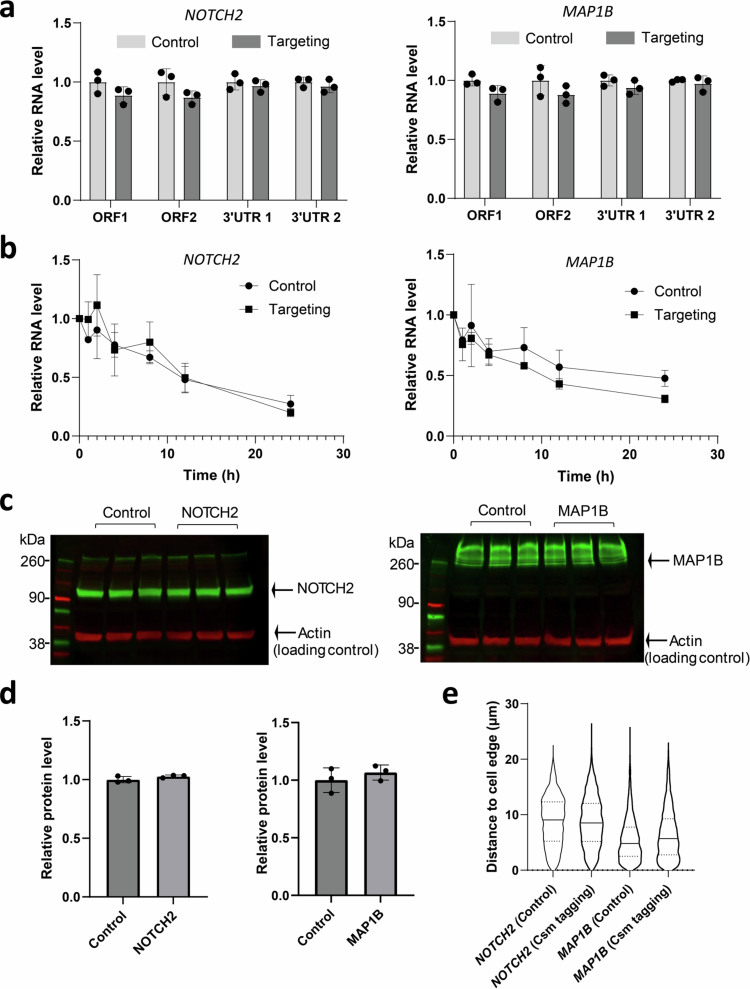
Quantification of RNA abundance, decay, protein level, and localization with or without Csm labeling. **a**, Relative abundance (normalized to *GAPDH*) of two ORF and two 3′ UTR regions of *NOTCH2* and *MAP1B* mRNAs, with (Targeting) and without (Control) Csm complex, as quantified by RT-qPCR. Error bars represent the mean ± standard deviation of three biological replicates. **b**, Relative abundance of *NOTCH2* (left) and *MAP1B* (right) mRNAs (normalized to *18S* rRNA) with (Targeting) or without (Control) Csm complex following transcription inhibition by Actinomycin D (at time 0). Error bars represent the mean ± standard deviation of three biological replicates. **c**, Western blot showing proper size and expression of NOTCH2 (left, green) and MAP1B (right, green) proteins with or without Csm complex. ACTB shown as loading control (red). Three biological replicates are shown. **d**, Quantification of protein bands in **c**. Error bars represent the mean ± standard deviation of three biological replicates. **e**, Violin plot showing distance to cell edge for individual *NOTCH2* or *MAP1B* mRNA molecules labeled using smFISH with or without Csm complex. Median indicated by solid line; quartiles indicated by dashed lines. Source data

Having established the efficacy of smLiveFISH, we asked whether labeling endogenous RNAs might perturb their activity in cells. Previous live-cell RNA imaging methods were found to alter target mRNA stability and/or localization^10,34,35^. Thus, we compared mRNA abundance, decay rate, localization and protein level for *NOTCH2* between Csm-labeled and unlabeled samples (Extended Data Fig. 7). Reverse transcription (RT)–qPCR measurements showed no significant change in steady-state mRNA levels across four different transcript regions (Extended Data Fig. 7a). We specifically examined both upstream and downstream of the Csm-tiled 3′ UTR region to check for accumulation of 5′ or 3′ degradation products, as has been reported for MS2 methods^10,34,35^. Furthermore, mRNA levels decayed at a similar rate following actinomycin D treatment, indicating no significant change in turnover time (Extended Data Fig. 7b). Western blotting showed invariable levels of NOTCH2 protein, suggesting that translation was unaffected (Extended Data Fig. 7c,d). Lastly, the location of mRNAs (identified by smFISH) was similar between Csm-labeled and unlabeled samples (Extended Data Fig. 7e). In summary, these results demonstrate smLiveFISH to be a robust, efficient and minimally invasive tool for visualizing unmodified endogenous RNAs at single-molecule resolution in many cell types.

### *NOTCH2* mRNAs display translation-dependent dynamics

**Fig. 2:**
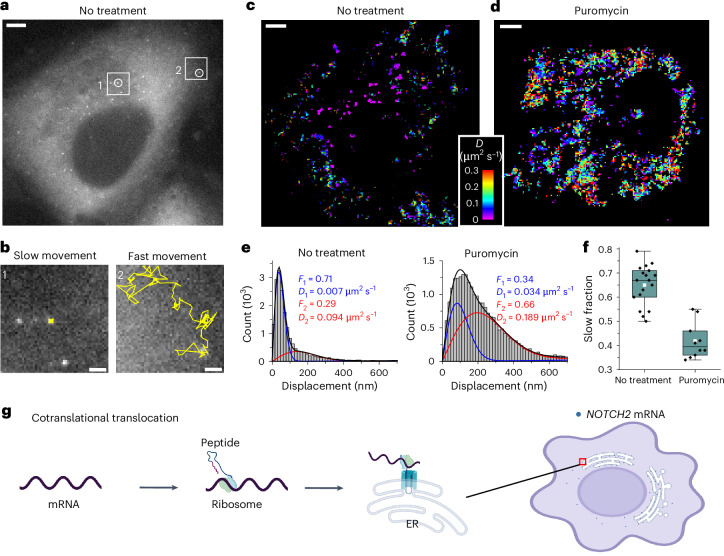
Dynamics of individual *NOTCH2* mRNAs in live cells. **a**, Single snapshot of individual *NOTCH2* mRNAs in live U2OS cell. Scale bar, 5 μm. **b**, Movement trajectories (yellow) over time of two highlighted mRNA foci in **a**, reflecting slow and fast movements. Images were recorded at ten frames per second over 25 s. Scale bar, 1 μm. **c**, Color-coded diffusivity map based on SMdM analysis of *NOTCH2* mRNA foci in the cell shown in **a**. **d**, Same as **c** but the cell was treated with puromycin for 1 h. The full video is shown in Supplementary Video 1. **e**, Distributions of single-molecule displacements across successive frames for the data shown in **c**,**d** (histograms) and fits (curves) to a two-component diffusion mode (Eq. (2)). Blue curve, slow component; red curve, fast component; black curve, sum. Resultant fractions of the two components and *D* values are marked in the plots. **f**, Quantification of the slow fraction in treated and untreated cells. Each data point corresponds to the analysis from one cell (no treatment, *n* = 18 cells from three biological replicates; puromycin treatment, *n* = 10 cells from three biological replicates). The box plots indicate the medians (center lines), means (white squares), first and third quartiles (bounds of boxes) and 1.5× the interquartile range (whiskers). **g**, A proposed cotranslational translocation mechanism directing *NOTCH2* mRNA to the cell perinuclear region by anchoring to the ER.

Next, we conducted live-cell imaging to examine the dynamics of *NOTCH2* mRNA in U2OS cells and observed two distinct mRNA populations according to their diffusion dynamics: slow-moving and fast-moving (Fig. 2a,b). We reasoned that anchoring to the ER membrane to allow cotranslational translocation^29,31^ of nascent polypeptide could explain why one population of *NOTCH2* mRNA was nearly static. To test this possibility, we treated cells with puromycin, a translation elongation inhibitor that causes release of mRNA from the nascent polypeptide^36^. Upon treatment, the static population of *NOTCH2* mRNA rapidly decreased (Fig. 2c,d and Supplementary Video 1). Single-molecule displacement and diffusivity mapping (SMdM) analysis^37^ of the two populations using a two-component diffusion mode (Eq. (2)) revealed that puromycin treatment correlated with a shift in the *NOTCH2* mRNA population from slow to fast movement (Fig. 2e,f). Taken together, these results suggest that stationary binding of *NOTCH2* transcripts to the perinuclear region is translation dependent, consistent with mRNA docking for peptide translocation across the ER (Fig. 2g).

### *MAP1B* mRNAs localize to the cell edge by directional transport

To explore the generalizability of smLiveFISH, we examined a second mRNA, *MAP1B*, which encodes a microtubule-associated protein involved in axon growth during neuronal development^38^. We hypothesized that *MAP1B* transcripts might use a distinct mechanism of transport because of their different spatial localization pattern relative to *NOTCH2* mRNA, as observed in fixed cells^30^.

**Fig. 3:**
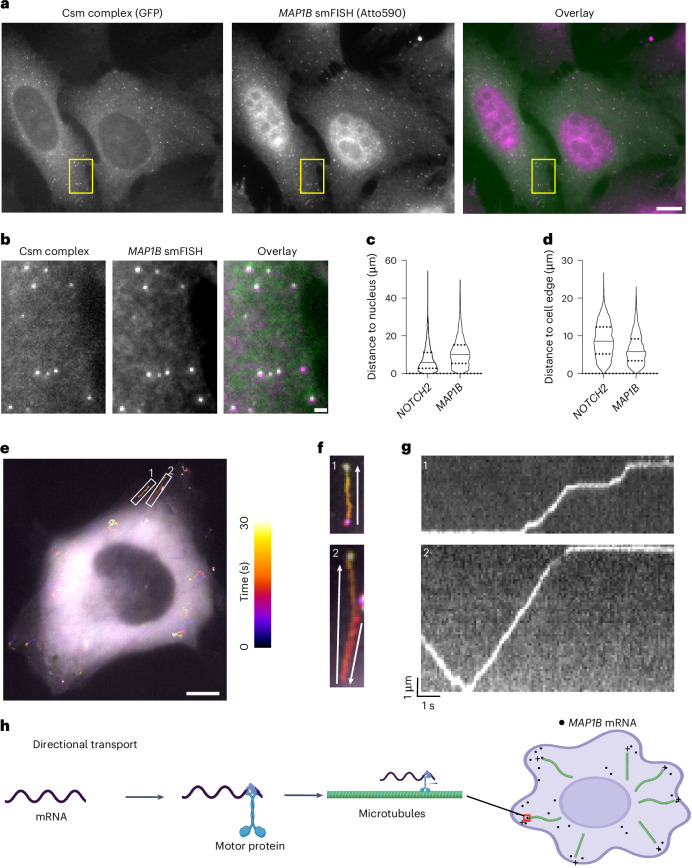
Dynamics of individual *MAP1B* mRNAs in live cells. **a**, Left, fixed-cell image of individual *MAP1B* mRNAs labeled by GFP-tagged Csm complex and 48 *MAP1B*-targeting crRNAs. Middle, image of individual *MAP1B* mRNAs labeled by smFISH probes. Right, overlaid image. Scale bar, 10 μm. **b**, Enlarged view of the yellow boxed region in **a**. Scale bar, 1 μm. **c**, Violin plot showing distance to the nucleus for individual *NOTCH2* or *MAP1B* mRNA molecules labeled with Csm complex. The median is indicated by the solid line; quartiles are indicated by the dashed lines. **d**, Same as **c** but showing distance to cell edge. **e**, Temporal color-coded trajectory of *MAP1B* mRNAs in live U2OS cell. Scale bar, 10 μm. White boxed regions highlight the directional transport of two *MAP1B* mRNA molecules. **f**, Enlarged views of the white boxed regions 1 and 2 in **e**. The full video is shown in Supplementary Video 2. White arrows indicate the direction of movement for each molecule. **g**, Kymograph of white boxed regions 1 and 2 in **e** showing directed movement of the indicated *MAP1B* mRNAs. **h**, A proposed mechanism for directional movement of *MAP1B* mRNA to the cell periphery through motor protein trafficking along microtubules.

We designed 48 crRNAs tiling the 3′ UTR of *MAP1B* mRNA (Supplementary Table 2) and transfected U2OS cells with Csm-complex-encoding and CRISPR-array-encoding plasmids. Similar to *NOTCH2* mRNA labeling, single-molecule spots were observed in the cytoplasm using GFP fluorescence detection, representing putative *MAP1B* mRNA signals (Fig. 3a,b). We validated these spots with separate *MAP1B* smFISH probes bearing a second color fluorophore and observed strong colocalization of Csm-labeled spots with smFISH spots (Fig. 3a,b). Using smLiveFISH, we investigated the spatial distribution of the labeled RNA species. By measuring the distance from mRNA molecules to the cell nucleus and/or cell edge, we found that *MAP1B* mRNAs were enriched at the cell periphery while *NOTCH2* mRNAs were enriched in the perinuclear region (Fig. 3c,d), in agreement with previous RNA FISH results in fixed cells^30^. To rule out potential interference with RNA behavior because of Csm labeling, we again compared mRNA abundance, decay rate, localization and protein levels of *MAP1B* between labeled and unlabeled samples and found no obvious differences (Extended Data Fig. 7).

To elucidate the mechanism that enriches *MAP1B* mRNAs at the cell periphery, we performed live-cell imaging. Strikingly, unlike *NOTCH2*, we frequently observed linear transport of *MAP1B* mRNAs toward the cell edge, upon which they remained relatively static (Fig. 3e–g and Supplementary Video 2). Occasionally, *MAP1B* mRNAs moved backward toward the cell nucleus but then eventually progressed again to the cell edge (for example, region of interest 2 in Fig. 3e–g and Supplementary Video 2). By analyzing these movement trajectories through kymograph, we also observed pausing of *MAP1B* mRNAs during transport (Fig. 3g and Supplementary Video 2). Previous RNA immunoprecipitation experiments demonstrated interactions between microtubule motor protein kinesin-1 and *MAP1B* mRNAs^39^. Taken together, these results suggest that, unlike perinuclear *NOTCH2* mRNA, *MAP1B* mRNA uses directional transport on microtubules as a driving force to localize to the cell edge (Fig. 3h).

### Translation inhibition sequesters *MAP1B* mRNA in P-bodies

Most transported mRNAs are thought to be translationally inactive until reaching their destination for local translation^40^ but a recent study showed that translation can also occur before or during transport^41^. Especially given that *MAP1B* mRNA encodes a microtubule-associated protein that may explain its transport along the cytoskeleton, we asked whether inhibiting translation influences its transit and/or postdestination dynamics.

**Fig. 4:**
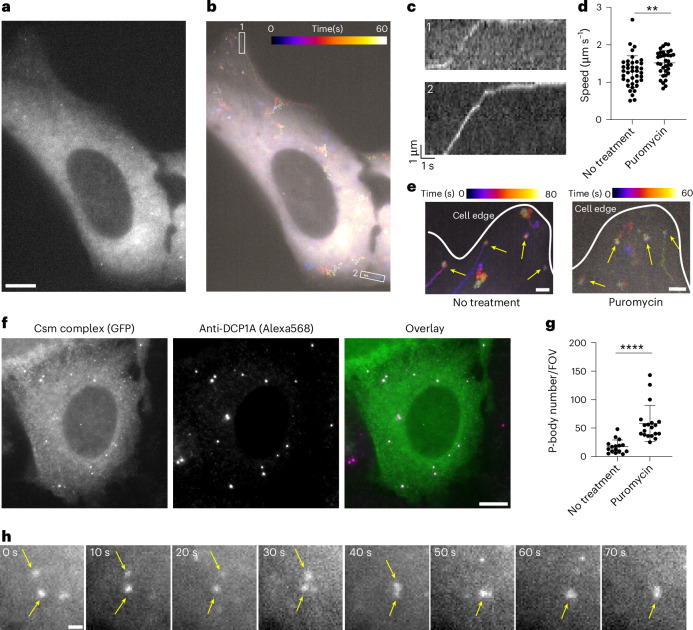
Dynamics of individual *MAP1B* mRNAs after puromycin treatment in live cells. **a**, Single snapshot of individual *MAP1B* mRNAs in live U2OS cell treated with puromycin. Scale bar, 10 μm. **b**, Temporal color-coded trajectory of *MAP1B* mRNAs in cell shown in **a**. **c**, Kymograph of white boxed regions 1 and 2 in **b** showing directed movement of the indicated *MAP1B* mRNAs. The full video is shown in Supplementary Video 3. **d**, Directed movement speeds of *MAP1B* mRNAs in untreated (*n* = 39 events) and puromycin-treated (*n* = 35 events) conditions. Each dot represents a directed movement event. Error bars represent the mean ± s.d. **P < 0.01 (two-tailed *t*-test). **e**, Temporal color-coded trajectory of *MAP1B* mRNAs at the cell edge. Yellow arrows mark stationary molecules. Scale bar, 2 μm. **f**, Left, fixed-cell image of *MAP1B* mRNAs labeled by GFP-tagged Csm complex after puromycin treatment. Middle, immunostaining for the P-body marker DCP1A. Right, overlaid image. Scale bar, 10 μm. **g**, Quantification of P-body number with or without puromycin treatment. Each dot represents the P-body number per field of view (FOV), with FOVs containing similar numbers of cells between conditions (no treatment, *n* = 16 FOVs; puromycin treatment, *n* = 19 FOVs). Error bars represent the mean ± s.d. ****P < 0.0001 (two-tailed *t*-test). **h**, Time-lapse micrographs of *MAP1B* RNA granule formation after puromycin treatment in live U2OS cell. Yellow arrows highlight two small puncta fusing into one larger granule. The full video is shown in Supplementary Video 4. Scale bar, 1 μm.

To test this, we treated cells with puromycin and performed smLiveFISH for *MAP1B* mRNA. The presence of puromycin did not change the observed directional transport of *MAP1B* mRNA toward the cell edge (Fig. 4a–c and Supplementary Video 3), suggesting that movement is not coupled to translation. In fact, the mean transit speed of *MAP1B* mRNAs increased slightly but significantly from 1.3 to 1.5 μm s^−1^ following puromycin treatment (Fig. 4d). This might have resulted from loss of polysomes and/or RBPs from *MAP1B* transcripts, a possibility that remains to be tested. Similarly, we observed no change in dynamics of the stationary *MAP1B* mRNA population already at the cell edge upon puromycin treatment compared to the obvious shift from slow to fast movement seen for *NOTCH2* mRNA (Fig. 4e and Supplementary Video 3). Thus, unlike *NOTCH2* mRNA, *MAP1B* mRNA localization dynamics are not translation dependent.

Interestingly, some *MAP1B* mRNAs coalesced into larger granules following puromycin treatment (Fig. 4f and Supplementary Video 3). Because puromycin treatment was shown to induce P-body formation and enlargement in U2OS cells^42^, we tested whether these RNA granules represent P-body formation. Costaining for the P-body marker DCP1A (mRNA decapping enzyme 1A) showed that P-body number increased after puromycin treatment and that *MAP1B* mRNA granules colocalized with P-bodies (Fig. 4f,g). We captured one example of two small RNA puncta moving randomly, contacting one another and eventually fusing into a single large granule (Fig. 4h and Supplementary Video 4). Together these data show that smLiveFISH can be used to study RNA storage and metabolism in living cells.

## Discussion

SmLiveFISH enables real-time tracking of unmodified endogenous mRNAs at single-molecule resolution, revealing both their spatial and their temporal dynamics in living cells. The platform uses two components, a Csm protein-encoding plasmid and a programmable crRNA-encoding plasmid. For the latter, we provide a generalizable method to construct long CRISPR arrays of up to 24 complementary sequences to a target RNA. Using smLiveFISH, we identified cotranslational translocation and directional transport as two distinct methods for *NOTCH2* and *MAP1B* mRNA localization to the perinuclear and peripheral regions of the cell, respectively.

SmLiveFISH represents a notable advance over previous live-cell RNA imaging techniques. It obviates the need for exogenous expression or genetic tagging of RNA^7–9^ and can be used to image low-abundance and nonrepetitive RNAs while maintaining single-molecule resolution and low background^3,4^. However, as with other live-cell RNA imaging methods, smLiveFISH may potentially alter native RNA behavior to some degree. Although we did not observe changes in mRNA stability (abundance, decay rate and degradation products) or translation, it is possible that tethering several Csm complexes to the 3′ UTR affects mRNA folding, binding of regulatory *trans*-factors (RBPs and microRNAs) or rates of transport and/or diffusion.

In parallel with smLiveFISH, we tested two Cas13 systems (PspCas13b and RfxCas13d) that have demonstrated imaging utility for highly abundant, repetitive RNAs^14,43^. Both Cas13 proteins had limited efficacy in our hands even when attempting to image the abundant and repetitive *XIST* long noncoding RNA (Extended Data Fig. 1). The comparative effectiveness of CRISPR–Csm for single-transcript imaging shown in this study may have resulted from Csm’s ~30-fold higher binding affinity for RNA compared to Cas13 (refs. ^23,27^) and/or its multisubunit nature, allowing for ≥3 Csm3–GFP molecules per complex.

In addition to benefits of using the Csm complex, smLiveFISH takes advantage of Cas6’s ability to process long pre-crRNAs, a property we harnessed to achieve single-molecule sensitivity. By tiling multiple crRNAs along an mRNA’s 3′ UTR, we were able to increase signal-to-noise ratio, with as few as 6–12 crRNAs sufficient for detection. It is important to note that smLiveFISH may have limited applicability to mRNAs with short UTR regions. Because approximately one of three spacers chosen at random has robust targeting efficiency (likely because of target secondary structure and availability)^15^, it is likely that fewer spacers could be used if they are selected using bioinformatic prediction methods^44^ or are first tested and verified for targeting efficiency. Lastly, we provide a step-by-step protocol for constructing large CRISPR arrays used in smLiveFISH imaging.

Mutations in RBPs contribute to multiple neurodegenerative disorders including mutated FMRP (fragile X mental retardation protein) in fragile X syndrome (FXS) and mutated TDP43 (TAR DNA-binding protein 43) in amyotrophic lateral sclerosis (ALS). Both FMRP and TDP43 are implicated in regulation of *MAP1B* mRNA transport, translation and/or stability in neurons^45–47^ and dysregulation of *MAP1B* and other target mRNAs is thought to contribute to disease^48^. SmLiveFISH can now be used as a sensitive assay to explore such mechanisms in ways that fixed-cell assays cannot, such as by measuring changes in mRNA transport speed or stepwise displacement (Figs. 3 and 4). These analyses may help uncover the pathological mechanisms of RNA-centric diseases such as FXS and ALS.

We observed different behaviors for *NOTCH2* and *MAP1B* mRNA after puromycin treatment, with *MAP1B* but not *NOTCH2* forming large RNA granules coincident with P-bodies. Previous sequencing results for mRNAs isolated from purified P-bodies support these findings^49^, whereby mRNAs encoding proteins involved in cell division, differentiation and morphogenesis are enriched in P-bodies, while those encoding integral ER proteins are depleted^49^. These findings suggest that *NOTCH2* and *MAP1B* mRNAs may rely on different decay pathways whose sorting mechanisms have yet to be elucidated.

Structural investigation of type III CRISPR–Cas systems indicates that the Csm1 and Csm4 subunits recognize the (−6) and (−7) nucleotides of the crRNA 5′ handle in a sequence-specific way^26^. Thus, it may be possible to develop orthogonal type III CRISPR–Cas systems with minimal crosstalk to the type III-A CRISPR–Csm complex from *S*. *thermophilus* used here, enabling multicolor live-cell RNA imaging. This method can then be extended to address questions about RNA–RNA interactions, splicing and cotranslational protein complex assembly. We expect smLiveFISH to accelerate efforts to study the spatiotemporal dynamics of various RNA species in many contexts, with immediate applications in the RNA, cell biology and neurobiology fields.

## Methods

### Cell lines and cell culture

HEK293T, U2OS, HeLa, IMR-90 and COS-7 cells were obtained from the University of California, Berkeley (UCB) cell culture facility and were grown in medium containing high-glucose DMEM (Thermo Fisher Scientific), 10% FBS (Sigma) and 1× penicillin–streptomycin (Thermo Fisher Scientific) at 37 °C with 5% CO_2_.

### Plasmid construction

The nucleus-targeting Csm complex plasmid construction was described previously^15^ (Addgene, plasmid 195242). A cytoplasm-targeting Csm complex plasmid was generated from this by removing the NLS sequences before each protein sequence, changing dCsm–EGFP to dCsm–2×sfGFP and removing the U6-crRNA region. dPspCas13b–3×EGFP plasmid was purchased from Addgene (plasmid 132398) and dRfxCas13d–EGFP was modified from Addgene plasmid 109050 by removing the T2A sequence between dRfxCas13d and EGFP.

For the CRISPR array plasmid, *NOTCH2* arrays were constructed through multiple steps of overlap extension PCR, as illustrated in Extended Data Fig. 2. Specifically, an oligo pool containing multiple 3–4-spacer fragments (sequences listed in Supplementary Table 1) was purchased from Integrated DNA Technologies (IDT). Then, the following steps were performed:

#### Step 1. Amplification of oligo pool fragments

A 50-µl PCR reaction was set up using Q5 high-fidelity 2× master mix (New England Biolabs (NEB)) containing 5 fmol of fragment (for example, spacers 1–4), 25 pmol of forward primer, 25 pmol of reverse primer, 25 µl of 2× master mix and water to a final volume of 50 µl. PCR was performed for 14–16 cycles. The PCR products were separated and purified from an agarose gel. If a smear was observed on the gel, the template amount or PCR cycle number was reduced.

#### Step 2. Joining of fragments

A 50-µl PCR reaction was set up using Q5 high-fidelity 2× master mix (NEB) containing 0.3 pmol of fragment 1 (for example, spacers 1–4 from step 1), 0.3 pmol of fragment 2 (for example, spacers 4–6 from step 1), 25 µl of 2× master mix and water to a final volume of 50 µl. Overlap extension PCR was performed for 6–8 cycles. The PCR products containing six spacers were separated and purified from an agarose gel.

#### Step 3. Addition of overhangs to joined fragments

A 50-µl PCR reaction was set up using Q5 high-fidelity 2× master mix (NEB) containing 5 fmol of joined fragment (for example, spacers 1–6 from step 2), 25 pmol of forward primer, 25 pmol of reverse primer (for example, containing overhang for spacer 7), 25 µl of 2× master mix and water to a final volume of 50 µl. PCR was performed for 14–16 cycles. The PCR products were separated and purified from an agarose gel.

#### Step 4. Joining of overhang-containing fragments

A 50-µl PCR reaction was set up using Q5 high-fidelity 2× master mix (NEB) containing 0.3 pmol of fragment 1 (for example, spacers 1–7 from step 3), 0.3 pmol of fragment 2 (for example, spacers 6–12 from step 3), 25 µl of 2× master mix and water to a final volume of 50 µl. Overlap extension PCR was performed for 6–8 cycles. The PCR products containing 12 spacers were separated and purified from an agarose gel.

#### Step 5. Cloning and sequence verification of intermediate fragments

The 12-spacer fragments from step 4 were cloned into vectors that were then introduced into bacteria for colony picking and sequence verification. Typically, 5–10 clones for each construct were sufficient to obtain a correct sequence.

#### Steps 6 and 7. Generation of final full-length arrays

Steps 3–5 were repeated to generate 24-spacer plasmids from the sequenced 12-spacer plasmids from step 5.

*MAP1B* arrays were constructed similarly but overhang regions were included in the original oligo pool sequences to bypass step 3 (Supplementary Table 1). Arrays were cloned downstream of a CAG promoter with a short signal sequence^28^ from the *HSPB3* gene placed before the array to enhance pre-crRNA export from the nucleus. All cloning was performed in NEB stable *Escherichia coli* (NEB) to prevent recombination between repetitive sequences. Plasmids were verified by whole-plasmid sequencing. CrRNA and oligo pool sequences are listed in Supplementary Table 1. Plasmid sequences are listed in Supplementary Table 2.

### Optical setup and image processing

Cell samples were imaged using a wide-field fluorescent microscope (Zeiss Axio Observer Z1 inverted fluorescence microscope) with a ×100/1.4 numerical aperture oil Ph3 Plan Apochromat objective, an ORCA-Flash4.0 camera (Hamamatsu), an X-Cite 120Q lamp and ZEN 2012 software. GFP filter sets included the BP 470/40 excitation filter, the FT 495 beamsplitter and the BP 525/50 emission filter. Atto590 and Alexa Fluor 568 filter sets included the BP 572/25 excitation filter, the FT 590 beamsplitter and the BP 629/62 emission filter. Images representing max-intensity *z*-projections were generated by FIJI software. Colocalization analysis was performed by FIJI plugin ComDet (version 0.5.5). Single-molecule tracking was performed by FIJI plugin TrackMate (version 7.12.1). The temporal color-coded images (Figs. 3e,f and 4b, e) were generated using the FIJI temporal color code function. Kymographs were generated using the KymoResliceWide plugin (version 0.6.0), with polyline selections used to track particle moving trajectories.

### Immunostaining

For H2AK119ub staining, 1.5 × 10^5^ HEK293T cells were grown on 18-mm-diameter, #1.5-thickness, collagen-coated coverslips (Neuvitro) in a 12-well plate. The next day, cells were transfected with 0.8 μg of *XIST-*targeting dCsm–GFP complex plasmid, 0.4 μg of dPspCas13b–3×EGFP plus 0.6 μg of *XIST*-targeting PspCas13b crRNA plasmid or 0.4 μg of dRfxCas13d–EGFP plus 0.6 μg *XIST-*targeting RfxCas13d crRNA plasmid using 5 μl of TransIT-293 transfection reagent (Mirus Bio). After transfection, cells were grown for 48 h to allow protein and crRNA expression. Then cells were fixed with 4% paraformaldehyde (Electron Microscopy Sciences) in 1× PBS at room temperature for 10–15 min. Following three washes with 1× PBS, cells were permeabilized by 0.5% (v/v) Triton X-100 (Sigma) in 1× PBS for 10 min at room temperature. Samples were again washed with 1× PBS three times after permeabilization. The permeabilized cells were incubated in a blocking buffer (1× PBS containing 3% (w/v) BSA (Jackson ImmunoResearch)) for 1 h. Cells were then incubated with anti-H2AK119ub primary antibodies at 1:1,000 dilution (Cell Signaling, 8240S) in blocking buffer for 1 h at room temperature and washed with 1× PBS three times for 5 min each. Next, cells were stained with Alexa Fluor 568-labeled secondary antibodies in blocking buffer for 1 h at room temperature. Samples were washed again with 1× PBS three times to remove unbound antibodies. To prevent bound antibody dissociation, samples were postfixed with 4% (v/v) PFA in 1× PBS for 10 min and washed three times with 1× PBS for 5 min each.

For DCP1A staining, 1 × 10^5^ U2OS cells were grown on 18-mm-diameter, #1.5-thickness, collagen-coated coverslips (Neuvitro) in a 12-well plate. The next day, 0.8 μg of cytoplasm-targeting Csm complex plasmid and two *MAP1B* CRISPR array plasmids (0.7 μg each) were transfected into cells using 5 μl of TransIT-LT1 transfection reagent (Mirus Bio). After transfection, cells were cultured for 48 h to allow protein and crRNA expression. Antibody staining was performed as for the above H2AK119ub procedure but with anti-DCP1A antibody (Abcam, ab183709).

### RNA FISH

HEK293T, HeLa, IMR-90 and COS-7 cells were grown on 18-mm-diameter, #1.5-thickness, collagen-coated coverslips (Neuvitro) in a 12-well plate. The next day, 0.8 μg of cytoplasm-targeting Csm complex plasmid and two CRISPR array plasmids (0.7 μg each) were transfected into cells using 5 μl of TransIT-293 transfection reagent (Mirus Bio) or TransIT-LT1 transfection reagent (Mirus Bio). For U2OS cells, 1 × 10^6^ cells were nucleofected with 1.5 μg of cytoplasm-targeting Csm complex plasmid and two CRISPR array plasmids (1.2 μg each). Then, U2OS cells were seeded on 18-mm-diameter, #1.5-thickness, collagen-coated coverslips (Neuvitro) at a density of 2.5 × 10^5^ cells per well. After transfection, cells were grown for 48 h to allow protein and crRNA expression, fixed with 4% paraformaldehyde (Electron Microscopy Sciences) and permeabilized with 0.5% (v/v) Triton X-100 (Sigma) in 1× PBS for 10 min at room temperature. After a 5-min incubation in wash buffer comprising 2× SSC (Thermo Fisher Scientific) and 30% (v/v) formamide (Thermo Fisher Scientific), cells were stained with *NOTCH2* or *MAP1B* mRNA FISH probes in hybridization buffer containing 30% (v/v) formamide, 0.1% (w/v) yeast tRNA (Thermo Fisher Scientific), 1% (v/v) murine RNase inhibitor (NEB), 10% (w/v) dextran sulfate (Sigma) and 2× SSC in a humidity-controlled 37 °C incubator overnight. FISH probes were applied at a concentration of 200 nM (5 nM per probe, ~40 probes in total). After staining, cells were washed twice with wash buffer at 37 °C, each for 30 min. Then, cells were stained with DAPI and 5 nM readout probes in a separate hybridization buffer composed of 2× SSC and 10% (v/v) ethylene carbonate (Sigma) in nuclease-free water before imaging. The *NOTCH2* and *MAP1B* mRNA FISH probe sequences and readout probe sequence are provided in Supplementary Table 3. *NOTCH2* and *MAP1B* FISH probes were ordered as oligo pools from IDT.

### Live-cell imaging

For live-cell imaging of *NOTCH2* and *MAP1B* mRNAs, 1 × 10^6^ U2OS cells were nucleofected with 1.5 μg of cytoplasm-targeting Csm complex plasmid and two CRISPR array plasmids (1.2 μg each). Then, U2OS cells were seeded in a two-well glass-bottom NuncLab-Tek chamber (Thermo Fisher Scientific) at a density of 4 × 10^5^ cells per well. After 48 h, the medium was changed to live-cell imaging buffer containing DMEM without phenol red supplied with 10% FBS, 1× penicillin–streptomycin and ProLong live antifade reagent (Thermo Fisher Scientific).

### Puromycin treatment

For puromycin treatment, cells were incubated in live-cell imaging buffer containing 275 μM puromycin for 60 min at 37 °C before fixation or live-cell imaging.

### Illustration software

Figures 2g and 3h were created using BioRender.com.

### SMdM data analysis

SMdM analyses were described previously^37^. Briefly, single-molecule spots were first localized in all frames. Paired locations were identified across successive frames for calculation of displacements in the frame time Δ*t* = 100 ms. The displacements were spatially binned with a grid size of 2.5 pixels (325 nm). The displacements in each spatial bin were separately fitted to a single-component diffusion mode through maximum likelihood estimation:

1 $$P(r)=\frac{2r}{a}\exp\left(-\frac{{r}^{2}}{a}\right)+br$$

Here, *a* = 4*D*Δ*t*, where *D* is the diffusion coefficient and *b* accounts for a uniform background. The resultant local apparent *D* values were presented on a continuous color scale to produce a diffusivity map (Fig. 2c,d). Separately (Fig. 2e,f), all single-molecule displacements in each cell were pooled and fitted to a two-component diffusion mode^50^:

2 $$P(r)={F}_{1}\frac{2r}{{a}_{1}}\exp\left(-\frac{{r}^{2}}{{a}_{1}}\right)+(1-{F}_{1})\frac{2r}{{a}_{2}\,}\exp\left(-\frac{{r}^{2}}{{a}_{2}}\right)+br$$

where *F*_1_ and *F*_2_ = (1 − *F*_1_) are the fractions of the two diffusivity components and *a*_1_ = 4*D*_1_Δ*t* and *a*_2_ = 4*D*_2_Δ*t* account for the two diffusion coefficients *D*_1_ and *D*_2_.

### RNA abundance measurements

Total cell RNA was extracted using TRIzol Reagent (Thermo Fisher Scientific) as per the manufacturer’s instructions. Genomic DNA was removed using TURBO DNase (Thermo Fisher Scientific). After inactivating TURBO DNase with DNase-inactivating reagent, 2 μg of DNase-free RNA was reverse-transcribed using SuperScript III reverse transcriptase (Thermo Fisher Scientific) with random primers (Promega) as per the manufacturer’s instructions. qPCR was performed using iTaq Universal SYBR green supermix (Bio-Rad) in a CFX96 real-time PCR detection system (Bio-Rad). Gene-specific primer pairs used to detect mature transcripts are listed in Supplementary Table 4. The relative amount of target RNA compared to *GAPDH* was calculated using the 2^−Δ^^Δ^^Ct^ method. Measurements were taken for three biological replicates, each with three technical replicates. No-RT and no-template controls were run alongside all RT–qPCR experiments.

### RNA decay measurement

Cells were treated with 10 µg ml^−1^ actinomycin D (Thermo Fisher Scientific) for 0, 1, 2, 4, 8, 12 and 24 h to block transcription. Then, total RNA was extracted using the Direct-zol MiniPrep kit (Zymo Research) according to the manufacturer’s instructions. qPCR was performed on a CFX96 real-time PCR detection system (Bio-Rad) with the one-step RT–qPCR Kit (Thermo Fisher Scientific) to determine relative RNA levels. The relative mRNA levels of *NOTCH2* and *MAP1B* versus the reference *18S* ribosomal RNA were determined using three biological replicates. Following PCR amplification, melting curve analysis confirmed a single PCR product for each target gene. PCR primer sequences are listed in Supplementary Table 4.

### Western blot

Cells were lysed in cold radioimmunoprecipitation assay lysis and extraction buffer (Thermo Fisher Scientific) supplemented with protease inhibitors (Sigma-Aldrich). Following centrifugation, the supernatants were collected and protein concentration measured using the Pierce 660-nm protein assay. Then, 10–30 µg of protein lysate was denatured in 1× Laemmli buffer at 95 °C for 10 min and resolved by SDS–PAGE. Proteins were transferred to an Immun-Blot LF PVDF membrane (Bio-Rad). The membrane was blocked with blocking buffer (0.05% Tween-20 and 3% BSA in 1× PBS) for 1 h at room temperature, incubated with primary antibody in blocking buffer for 2 h at room temperature, washed three times with 1× PBS, incubated with dye-conjugated secondary antibody for 1 h at room temperature and washed three more times with 1× PBS. The 700-nm and 800-nm channels of a LI-COR Odyssey CLx were used to visualize protein bands. The following primary antibodies were used for western blot: anti-NOTCH2 (Cell Signaling, 5732S; 1:1,000 dilution), anti-MAP1B (Thermo Fisher Scientific, PA5-82798; 1:1,000 dilution) and anti-ACTB (Proteintech, 60008-1-Ig; 1:2,500 dilution). The following secondary antibodies were used: IRDye 680RD goat anti-mouse (LI-COR, 926-68070; 1:20,000 dilution) and IRDye 800CW goat anti-rabbit (LI-COR, 926-32211; 1:20,000 dilution). Fiji was used to quantify the relative band intensities on blot images.

### Statistics and reproducibility

Statistical analyses were conducted using GraphPad Prism (version 10.2.2). Exact statistical values are presented in the figures. The microscopy images presented from representative experiments were independently replicated at least three times with similar outcomes, unless explicitly indicated by the sample size noted in each figure.

### Reporting summary

Further information on research design is available in the Nature Portfolio Reporting Summary linked to this article.

## Online content

Any methods, additional references, Nature Portfolio reporting summaries, source data, extended data, supplementary information, acknowledgements, peer review information; details of author contributions and competing interests; and statements of data and code availability are available at 10.1038/s41587-024-02540-5.

## Supplementary information

Supplementary Information Supplementary Tables 1–4.

Reporting Summary

Supplementary Video 1 *NOTCH2* mRNA visualized 60 min after addition of puromycin. Compared to no treatment (left), movement of *NOTCH2* mRNA shifts from slow-moving to fast-moving. Video recorded at ten frames per second. Scale bar, 5 μm.

Supplementary Video 2 Live-cell imaging of *MAP1B* mRNA in untreated U2OS cells. Video recorded at ten frames per second. Scale bar, 10 μm.

Supplementary Video 3 Live-cell imaging of *MAP1B* mRNA in puromycin-treated U2OS cells. Video recorded at ten frames per second. Scale bar, 10 μm.

Supplementary Video 4 Live-cell imaging of *MAP1B* mRNA in puromycin-treated U2OS cells. The white boxed region shows *MAP1B* RNA granule formation. Video recorded at ten frames per second. Scale bar, 10 μm.

## Source data

Source Data Extended Data Fig. 7 Unprocessed western blots.

## Data Availability

Oligo and plasmids sequences in this study are available in the Supplementary Information. Essential plasmids were deposited to Addgene (plasmids 229211–229216). Unprocessed microscope image files are available through figshare^51^ (10.6084/m9.figshare.27997130). Source data are provided with this paper.

## Code availability

All codes are available upon request.
