# Supplementary material for: Single-molecule live-cell RNA imaging with CRISPR–Csm
Source: Nat Biotechnol. 2025 Feb 18;43(12):2023–30. doi: 10.1038/s41587-024-02540-5 (PMC12700784; doi:10.1038/s41587-024-02540-5)
Supplement: Supplementary file 1 — Supplementary Tables 1–4. [file 41587_2024_2540_MOESM1_ESM.pdf]

nature biotechnology

Supplementary information https://doi.org/10.1038/s41587-024-02540-5

# Single-molecule live-cell RNA imaging with CRISPR–Csm

In the format provided by the authors and unedited

## Supplementary Table 1. crRNA and oligo pool sequences.

| CRISPR-Cas system | XIST crRNA spacer sequence |
|---|---|
| PspCas13b | AAAGCAGGTATCCGCGGCCCCG |
| RfxCas13d | AAAGCAGGTATCCGCGGCCCCG |
| Csm complex | AAAAGCAGGTATCCGCGGCCCCGATGGGCAAA |

| Oligo name | Oligo sequences |
|---|---|
| TypeIIIA_array_NOTCH2_pool1_segment1 | atcgaatccttaagctacgaacgatataaacctaattacctcgagaggggacggaaacTTTGTTCCTCAGCAGCATTTACAAAAGTCAGTTATGgatataaacctaattacctcgagaggggacggaaacTACCCAGTGAAACTGACGAATTGCTTCTCTTGCATTgatataaacctaattacctcgagaggggacggaaacTACCTCTAGAAGCTGGCTCCAGAGATTTCTTCATTTgatataaacctaattacctcgagaggggacggaaacTTGTATTCATCTTGCATTTCCACAA |
| TypeIIIA_array_NOTCH2_pool1_segment2 | TTGTATTCATCTTGCATTTCCACAAACTTGTCTTATgatataaacctaattacctcgagaggggacggaaacTTCAATAAGCATCCATCTTATTCTCCAAATAGAAGAgatataaacctaattacctcgagaggggacggaaacTTCTCATGGATATGGCAGAAGCCTGCAGGGCTTAAAgatataaacctaattacctcgagaggggacggaaacAGCGCAATTACGCTTACGGTG |
| TypeIIIA_array_NOTCH2_pool1_segment3 | tcgtgcacagtaggatataagcgatataaacctaattacctcgagaggggacggaaacTTTCATGGGCCAGAATGATCAAAAGCACAAAGGTCTgatataaacctaattacctcgagaggggacggaaacTTTATATGAAGACCTGCACACAGACAAGAAAAATTAgatataaacctaattacctcgagaggggacggaaacTTTGTGCTGAGAACCATACCAAAGTAAACCTTGTGAgatataaacctaattacctcgagaggggacggaaacTTCTCTCTCCAGGAATGATATATGC |
| TypeIIIA_array_NOTCH2_pool1_segment4 | TTCTCTCTCCAGGAATGATATATGCAGGAGAACACAgatataaacctaattacctcgagaggggacggaaacTTCCAAACCTTTTTCTGGTTTTAAAAAAGTGGGGAGgatataaacctaattacctcgagaggggacggaaacTTTACACTGACCAGGCCCATACACAGAAAAACAACAgatataaacctaattacctcgagaggggacggaaacTCTCACGTACGACTGATGCA |
| TypeIIIA_array_NOTCH2_pool1_segment5 | acctacgttgatgtatctctcagatataaacctaattacctcgagaggggacggaaacAAACAGGCCTAGAATGCTCAGATCTGGGACAAATGTgatataaacctaattacctcgagaggggacggaaacTTGGCAGGAAGTAGGGGGACCTGTGGGTGGGTAACTgatataaacctaattacctcgagaggggacggaaacTTCTGTGGATATGAAAGGAAAGGCTCAACAGTTAAGgatataaacctaattacctcgagaggggacggaaacTTGGTGAGCAGGCCTTTAAGCAGCA |
| TypeIIIA_array_NOTCH2_pool1_segment6 | TTGGTGAGCAGGCCTTTAAGCAGCAAGATAATCAGTgatataaacctaattacctcgagaggggacggaaacTGTCACTGGGTCCCTTGGGCTAAGGAAAGTTCAGTCgatataaacctaattacctcgagaggggacggaaacTATAAAGTCCATGTCTTCAGTGAGAACATACTGGGTgatataaacctaattacctcgagaggggacggaaacCGCTTAAGCAGATTGGTCCA |
| TypeIIIA_array_NOTCH2_pool1_segment7 | taccacgcaatagtatccatcggatataaacctaattacctcgagaggggacggaaacTTCTTATAGGGCTGTTTTGGATCATAGAAAACAAGTgatataaacctaattacctcgagaggggacggaaacTAGGAAAGGAAAATGTTCTGTTGAGTTTCTACGTTAgatataaacctaattacctcgagaggggacggaaacTTCAGAGGAAAAGAAATGGTAGCAGAAAATAGCAAAgatataaacctaattacctcgagaggggacggaaacTTCAGGTGAGGGGCAATCAGTCTGA |
| TypeIIIA_array_NOTCH2_pool1_segment8 | TTCAGGTGAGGGGCAATCAGTCTGAACAATGAGCAAgatataaacctaattacctcgagaggggacggaaacTTAACTAAAATGCTTCTGCCCTTAAAAGAAAGTCAAgatataaacctaattacctcgagaggggacggaaacTTGCAGTGTAGCCATGGACCAATTATTAACTGGCCCgatataaacctaattacctcgagaggggacggaaacAGGTTAACACTGCCCTTGAA |
| TypeIIIA_array_NOTCH2_pool2_segment1 | atcgaatccttaagctacgaacgatataaacctaattacctcgagaggggacggaaacAAAGAGTCGGGAATTCACCTGTTAATATCTACAAAAgatataaacctaattacctcgagaggggacggaaacATAGGACTGAAAAAACCATTTGTAAACTATTATGCCgatataaacctaattacctcgagaggggacggaaacAAATCAGGTAAGTGGGAAGCACTGATGCATACTTGCgatataaacctaattacctcgagaggggacggaaacAAATGGGTCCAAGGGCAGAGAGTCA |
| TypeIIIA_array_NOTCH2_pool2_segment2 | GTGGGAAGCACTGATGCATACTTGCgatataaacctaattacctcgagaggggacggaaacAAATGGGTCCAAGGGCAGAGAGTCACCATTTAGAATgatataaacctaattacctcgagaggggacggaaacAAAGCAGAGAATGAACAAACTTAATCTGCTTATCTCgatataaacctaattacctcgagaggggacggaaacAGGGCAGAAAGGGGCAGGTACGCTGTGGTCCACAGAgatataaacctaattacctcgagaggggacggaaacAGCGCAATTACGCTTACGGTG |
| TypeIIIA_array_NOTCH2_pool2_segment3 | tcgtgcacagtaggatataagcgatataaacctaattacctcgagaggggacggaaacAAAAGGGAGGATATAAAATATCCAGTGGCTGGATCAgatataaacctaattacctcgagaggggacggaaacATGGCATAAAAGATGTGTCTCATAAAAACTGAGTCTgatataaacctaattacctcgagaggggacggaaacAAAGGCAGTGTTGTCCTCCTCATCACTGAAACCTGTgatataaacctaattacctcgagaggggacggaaacAAACCCAAAGGATCCAAATTAAAGT |

| | |
|---|---|
| TypeIIIA_array_NOTCH2_pool2_segment4 | TGTCCTCCTCATCACTGAAACCTGTgatataaacctaattacctcgagaggggacggaaacAAACCCAAAGGATCCAAATTAAAGTAGTCCAAGAAAgatataaacctaattacctcgagaggggacggaaacTTACCTTCCCTTTTATTAGATACCACACAATTCAAGgatataaacctaattacctcgagaggggacggaaacAGAAGAGGAGTCAAAGAAAGAAAGCATTTATAACAAgatataaacctaattacctcgagaggggacggaaacTCTCACGTACGACTGATGCA |
| TypeIIIA_array_NOTCH2_pool2_segment5 | acctacgttgatgtatctctcagatataaacctaattacctcgagaggggacggaaacACCTCACATAAGAAAATGATGCTTAAAACAAAACAGgatataaacctaattacctcgagaggggacggaaacAAAACATTACACCTTTGGTTCTTTATTATGCAAAAAgatataaacctaattacctcgagaggggacggaaacTTTGAGCATCACAGCCAATTGCTTATACTAAAATATgatataaacctaattacctcgagaggggacggaaacAAATGTGTCCTCCCAAATAGTCATC |
| TypeIIIA_array_NOTCH2_pool2_segment6 | CACAGCCAATTGCTTATACTAAAATATgatataaacctaattacctcgagaggggacggaaacAAATGTGTCCTCCCAAATAGTCATCATACATTCAATgatataaacctaattacctcgagaggggacggaaacAAAGTGTGCATTTTCCTTACTACGTTTAGTCAGGAAgatataaacctaattacctcgagaggggacggaaacATATCACTTTTAAGAGAAATGTACACAAGGAAGTAAgatataaacctaattacctcgagaggggacggaaacCGCTTAAGCAGATTGGTCCA |
| TypeIIIA_array_NOTCH2_pool2_segment7 | taccacgcaatagtatccatcggatataaacctaattacctcgagaggggacggaaacTTTTGTGATAAAAATGCTTTCATATAAATTTCATCTgatataaacctaattacctcgagaggggacggaaacTTTGTGGGATTCAGAAAGAAAAGAAAATTTGCTCTCgatataaacctaattacctcgagaggggacggaaacTGCCAGGCCATGGATGCAGTATTGGAAAATAAAAAAgatataaacctaattacctcgagaggggacggaaacTTTCCCCAGGATCATTTATTTATGA |
| TypeIIIA_array_NOTCH2_pool2_segment8 | ATGGATGCAGTATTGGAAAATAAAAAAgatataaacctaattacctcgagaggggacggaaacTTTCCCCAGGATCATTTATTTATGATCTAATTAAAGgatataaacctaattacctcgagaggggacggaaacTTCCCAATTCTTGCTATAGCAACTACTTCGCATTTCgatataaacctaattacctcgagaggggacggaaacTTTGTCACCAGCTATGGCTAGTGCACAGAAGAGTGCgatataaacctaattacctcgagaggggacggaaacAGGTTAACACTGCCCTTGAA |
| TypeIIIA_array_MAP1B_segment1 | atcgaatccttaagctacgaacgatataaacctaattacctcgagaggggacggaaacTTTCTGGAAACAAAGTTCAGATCCTGTGGTGTgatataaacctaattacctcgagaggggacggaaacCCTTAGCAATCAGCAAAAACTGAGAAATTTAGgatataaacctaattacctcgagaggggacggaaacCAGGTAATTGTTCAGCGACTTAACTAGATGAAgatataaacctaattacctcgagaggggacggaaacGAGATGAACTGGGGTAGGTAATTTTGCAGGAA |
| TypeIIIA_array_MAP1B_segment2 | GAGATGAACTGGGGTAGGTAATTTTGCAGGAAgatataaacctaattacctcgagaggggacggaaacGACAAATGCCTCTACCTAGCAGAAGTCAGTTGgatataaacctaattacctcgagaggggacggaaacTCTCTCCTGCGTTATCTTTGTGCTCTCACTCTgatataaacctaattacctcgagaggggacggaaacTCACCAGAACTCTCTGTCATTACATCTCCTTCgatataaacctaattacctcgagaggggacggaaacACTGACAAAAATGGAAAACTTTGCC |
| TypeIIIA_array_MAP1B_segment3 | TCACCAGAACTCTCTGTCATTACATCTCCTTCgatataaacctaattacctcgagaggggacggaaacACTGACAAAAATGGAAAACTTTGCCTATGTAGgatataaacctaattacctcgagaggggacggaaacCCTAATTGTTGGTTTATTTTCTCCTCCTTACCgatataaacctaattacctcgagaggggacggaaacGTTGTTCTTTTTACTTTCTTCTAGCCAACTTG |
| TypeIIIA_array_MAP1B_segment4 | GTTGTTCTTTTTACTTTCTTCTAGCCAACTTGgatataaacctaattacctcgagaggggacggaaacGTGTGCAGCCTTCAAACTGAAGGAGCTTAATTgatataaacctaattacctcgagaggggacggaaacGTCTGAGAGTTCTGAATGGGGCCCAAACCACTgatataaacctaattacctcgagaggggacggaaacAGCGCAATTACGCTTACGGT |
| TypeIIIA_array_MAP1B_segment5 | tcgtgcacagtaggatataagcgatataaacctaattacctcgagaggggacggaaacCAACTGCTTTGGTATTTGCTTCAGTTAATTGGgatataaacctaattacctcgagaggggacggaaacTCAGAGTGGACTAAGTGGGTGGACAAATGCTGgatataaacctaattacctcgagaggggacggaaacAAGTGCATCAAGTATCTTTGTACATTCAAGTCgatataaacctaattacctcgagaggggacggaaacCATGACTCAGCATCAAACTGTTGGACCAATCA |
| TypeIIIA_array_MAP1B_segment6 | CATGACTCAGCATCAAACTGTTGGACCAATCAgatataaacctaattacctcgagaggggacggaaacCGTGTTCCTTTAAGTATCTATAATCCTATATGgatataaacctaattacctcgagaggggacggaaacAAGCTGTGAAAGGCACAACTTGTTTGACTGAGgatataaacctaattacctcgagaggggacggaaacAAGATTCACTGTTTTCCCAGTTCTGATCATAGgatataaacctaattacctcgagaggggacggaaacAAGCCAATGTAAGACAAAGGAAGG |
| TypeIIIA_array_MAP1B_segment7 | AAGATTCACTGTTTTCCCAGTTCTGATCATAGgatataaacctaattacctcgagaggggacggaaacAAGCCAATGTAAGACAAAGGAAGGTAAATACTgatataaacctaattacctcgagaggggacggaaacTAAATGAAAACATTTGTTAAAAAGTTCATCTAgatataaacctaattacctcgagaggggacggaaacTCTTCTGTACACTTGTTCCCATAATTATGTTG |

| | |
|---|---|
| TypeIIIA_array_MAP1B_segment8 | TCTTCTGTACACTTGTTCCCATAATTATGTTGgatataaacctaattacctcgagaggggacggaaacCATTATTCAATTTTTGTCTTACGAAACACTTAgatataaacctaattacctcgagaggggacggaaacGTTATGGGTTTTTAATAAATATCATTACTGCAgatataaacctaattacctcgagaggggacggaaacTCTCACGTACGACTGATGCA |
| TypeIIIA_array_MAP1B_segment9 | acctacgttgatgtatctctcagatataaacctaattacctcgagaggggacggaaacTCTCCCCCACTCTCAAGTAGCACTTTTATGTTgatataaacctaattacctcgagaggggacggaaacAGAAGTATGTAGGATACCACTGGGTGATGTACgatataaacctaattacctcgagaggggacggaaacGAACCGTAGGAGTTGAGAATTCATTTGTTTTCgatataaacctaattacctcgagaggggacggaaacGAAAATCCCAAAGCAAATTTGTGCTAGGGTTC |
| TypeIIIA_array_MAP1B_segment10 | GAAAATCCCAAAGCAAATTTGTGCTAGGGTTCgatataaacctaattacctcgagaggggacggaaacTAATCATGATCTGGCTTCCCAGGTTCACAGTTgatataaacctaattacctcgagaggggacggaaacACGGAGAAGTTTGCCTCATCTGAACACTGGGTgatataaacctaattacctcgagaggggacggaaacTCATTGTTCTCTACTCCGTAGATCTTAGAGTCgatataaacctaattacctcgagaggggacggaaacGACTTATCAGACAAAAATCAAC |
| TypeIIIA_array_MAP1B_segment11 | TCATTGTTCTCTACTCCGTAGATCTTAGAGTCgatataaacctaattacctcgagaggggacggaaacGACTTATCAGACAAAAATCAACTAAAATGTTAgatataaacctaattacctcgagaggggacggaaacTAGGTCCTCAGTGAAAGGACCCTGAAGAAGCAgatataaacctaattacctcgagaggggacggaaacTTTCCAAGGACTTGAAAGAAATGGGGTAATAA |
| TypeIIIA_array_MAP1B_segment12 | TTTCCAAGGACTTGAAAGAAATGGGGTAATAAgatataaacctaattacctcgagaggggacggaaacATATTAATTTTCTTTAAAAAATTAAACATTTGgatataaacctaattacctcgagaggggacggaaacACTCAATGAAATTCATTGGCGTCACAATGACTgatataaacctaattacctcgagaggggacggaaacCGCTTAAGCAGATTGGTCCA |
| TypeIIIA_array_MAP1B_segment13 | taccacgcaatagtatccatcggatataaacctaattacctcgagaggggacggaaacCCATTAAATAATTATCTTGAGTATCTTAGGTTgatataaacctaattacctcgagaggggacggaaacACATTTAATTGTGAAATTGGAAAATAACTTGCgatataaacctaattacctcgagaggggacggaaacTTGGCAAGAAGGCTAAAAATCTGGTTTTCTTCgatataaacctaattacctcgagaggggacggaaacAACTGAAGACTGGCTATAATTCTCCTACAATG |
| TypeIIIA_array_MAP1B_segment14 | AACTGAAGACTGGCTATAATTCTCCTACAATGgatataaacctaattacctcgagaggggacggaaacTTTACATATTTTTCCCATTAAATCCCATAGCAgatataaacctaattacctcgagaggggacggaaacTGAGAACAACGGATGGAGAAAGGTACTGATAGgatataaacctaattacctcgagaggggacggaaacTGGAAGGACAGGAGCAACCTGTAATGCGAATAgatataaacctaattacctcgagaggggacggaaacGGCTGTTACTAAACTAATCGTG |
| TypeIIIA_array_MAP1B_segment15 | TGGAAGGACAGGAGCAACCTGTAATGCGAATAgatataaacctaattacctcgagaggggacggaaacGGCTGTTACTAAACTAATCGTGTATTTATGGAgatataaacctaattacctcgagaggggacggaaacTGAACAAAGCTCCTAGAAGTCTGTGTCGATCTgatataaacctaattacctcgagaggggacggaaacCATTTAAACAGAGTGTAAATTCCTTCCACTCA |
| TypeIIIA_array_MAP1B_segment16 | CATTTAAACAGAGTGTAAATTCCTTCCACTCAgatataaacctaattacctcgagaggggacggaaacCTCTGTAGAATTTTGTTTAAGAATACTACCAAgatataaacctaattacctcgagaggggacggaaacCTCAGGGTTTTGAGAAAGGGGACAGAATTGGTgatataaacctaattacctcgagaggggacggaaacAGGTTAACACTGCCCTTGAA |

**Supplementary Table 2. Csm complex and CRISPR array plasmid sequences.**

| | |
|---|---|
| Plasmid | pCX420 |
| Description | Cytoplasmic-targeting Csm complex plasmid |

GTGATGCGGTTTTGGCAGTACATCAATGGGCGTGGATAGCGGTTTGACTCACGGGGATTTCCAAGTCTCCACCCCATTGACGTCAATGGGAGTTT
GTTTTGGCACCAAAATCAACGGGACTTTCCAAAATGTCGTAACAACTCCGCCCCATTGACGCAAATGGGCGGTAGGCGTGTACGGTGGGAGGTCT
ATATAAGCAGAGCTCGTTTAGTGAACCGTCAGATCTCTAGAgccgccaccATGCACCATCACCATCACCATTCCGGCGATTACAAAGACGATGACGATA
AGCGGGGCATGAAGAAAGAAAAGATTGATCTGTTTTACGGAGCCCTGCTGCACGACATCGGAAAGGTCATCCAGCGAGCAACCGGAGAGCGGAA
GAAACACGCACTTGTGGGCGCCGACTGGTTCGACGAGATCGCCGACAACCAAGTCATCTCGGATCAGATCCGGTACCATATGGCCAACTACCAGT
CTGATAAGCTCGGCAACGATCACCTGGCTTACATCACCTACATTGCCGACAACATCGCCTCCGGTGTCGACCGCCGGCAATCCAACGAAGAGTCA
GACGAAGATACCTCCGCAAAGATCTGGGACACCTACACGAACCAGGCCGACATCTTTAACGTGTTCGGAGCGCAGACCGATAAGCGGTACTTCAA
GCCTACCGTGCTGAATCTCAAGTCGAAGCCCAACTTCGCGTCCGCCACTTACGAACCCTTTAGCAAGGGCGATTACGCTGCCATCGCCACCCGGA
TTAAGAACGAACTGGCCGAGTTCGAGTTCAACCAAGTCCAGATTGACTCCCTGCTCAACCTTTTCGAGGCTACTCTCTCCTTCGTGCCGTCAAGCA
CCAACACTAAGGAAATCGCCGACATCTCCCTGGCCGACCATTCCCGCTTGACTGCTGCCTTCGCTCTGGCGATCTACGACTACCTGGAGGACAAG
GGTCGGCACAACTACAAAGAGGACCTGTTCACCAAAGTGTCAGCGTTCTATGAAGAAGAAGCCTTCCTGCTGGCCTCCTTCGACCTGTCGGGAAT
CCAGGACTTTATCTACAACATTAACATCGCAACTAACGGCGCGGCGAAGCAGCTGAAGGCCCGGAGCCTCTACCTGGACTTTATGTCCGAGTACA
TCGCCGATAGCCTGCTGGACAAGCTGGGACTGAACAGGGCTAACATGCTTTACGTCGGCGGCGGACACGCCTACTTCGTCCTGGCCAACACCGA
AAAGACTGTGGAAACCCTGGTGCAGTTTGAGAAGGATTTCAACCAGTTCCTGTTGGCAAACTTCCAGACCCGCCTCTATGTGGCCTTTGGCTGGG
GTTCCTTCGCGGCCAAGGACATCATGTCCGAGCTGAATAGCCCCGAGTCCTACCGCCAAGTGTACCAAAAGGCTTCGCGCATGATCTCCAAAAAG
AAAATCTCCAGATACGACTACCAGACACTGATGCTCCTGAATCGCGGTGGAAAGTCCTCAGAGAGAGAGTGCGAGATTTGCCACTCCGTGGAGAA
CCTGGTGTCCTACCACGACCAGAAAGTCTGTGACATTTGCCGGGGACTGTACCAGTTCTCGAAAGAAATTGCCCATGACCACTTCATCATTACCGA
AAATGAGGGGCTGCCGATTGGACCAAACGCGTGCTTAAAGGGCGTGGCATTCGAAAAGCTGTCCCAAGAAGCGTTCAGCCGGGTCTACGTGAAG
AATGACTATAAGGCCGGTACCGTGAAGGCTACGCATGTGTTCGTGGGGGATTACCAGTGCGACGAGATCTACAACTACGCCGCCCTGAGCAAGAA
CGAGAACGGCCTAGGCATCAAGAGACTGGCCGTGGTCCGGCTCGACGTGGATGACTTGGGCGCCGCCTTCATGGCCGGTTTCAGCCAGCAGGG
AAACGGACAATACTCCACTCTGTCAAGATCGGCCACATTCTCCCGGAGCATGTCGCTGTTCTTCAAAGTGTACATTAACCAGTTCGCCTCCGACAA
GAAGCTGAGCATTATCTACGCGGGCGGCGATGACGTGTTCGCCATTGGATCGTGGCAGGATATCATCGCGTTCACTGTGGAACTTCGCGAAAACT
TCATCAAGTGGACCAACGGGAAGCTCACCCTCTCCGCGGGGATAGGGTTGTTCGCCGACAAGACTCCTATTAGCCTGATGGCTCACCAGACCGG
GGAACTGGAAGAGGCCGCCAAGGGCAACGAAAAGGACTCCATCTCGCTGTTCTCAAGCGACTACACTTTCAAGTTTGATAGGTTCATCACTAACGT
GTACGACGACAAACTGGAACAGATTAGATACTTCTTCAACCATCAAGACGAGAGGGGAAAGAACTTCATCTATAAGCTTATTGAGCTTTTGAGGAAC
CACGACCGCATGAATATGGCACGCCTCGCCTATTACCTCACTCGCCTGGAAGAACTGACCCGGGAGACTGACAGGGACAAGTTCAAGACCTTCAA
GAACCTGTTCTACTCCTGGTACACCAACAAGAACGATAAGGACCGGAAGGAAGCCGAGCTCGCGCTCCTGCTGTACATCTACGAAATCAGAAAGG
ATTAAcggcaataaaaagacagaataaaacgcacggtgttgggtcgtttgttcGCACACATTAGCTAGCCGTCAGCACACATTGTGATGCGGTTTTGGCAGTACATCAATG
GGCGTGGATAGCGGTTTGACTCACGGGGATTTCCAAGTCTCCACCCCATTGACGTCAATGGGAGTTTGTTTTGGCACCAAAATCAACGGGACTTTC
CAAAATGTCGTAACAACTCCGCCCCATTGACGCAAATGGGCGGTAGGCGTGTACGGTGGGAGGTCTATATAAGCAGAGCTCGTTTAGTGAACCGT
CAGATCTCTAGAgccgccaccATGGATTACAAAGACGATGACGATAAGCGGGGCATGACCATCCTGACCGACGAGAACTACGTGGACATCGCCGAGA
AAGCCATCCTGAAGCTGGAAAGAAACACCAGAAATAGAAAGAACCCTGATGCCTTCTTCCTGACCACATCTAAGCTGCGGAACCTGCTGAGCCTGA
CAAGCACCCTGTTCGACGAGAGCAAGGTGAAGGAATACGACGCCCTGCTGGACAGAATCGCTTATCTGAGAGTGCAGTTCGTGTACCAGGCCGG
CAGAGAGATCGCCGTGAAAGATCTGATCGAGAAGGCCCAGATCCTGGAAGCTCTGAAAGAGATCAAGGACCGGGAAACCCTGCAGAGATTCTGC
AGATACATGGAAGCCCTGGTGGCCTACTTCAAGTTCTACGGCGGCAAGGACTGAcggcaataaaaagacagaataaaacgcacggtgttgggtcgtttgttcAGTTCTTT
TGCCTTACTTTCAATGCATGCGGTGATGCGGTTTTGGCAGTACATCAATGGGCGTGGATAGCGGTTTGACTCACGGGGATTTCCAAGTCTCCACCC
CATTGACGTCAATGGGAGTTTGTTTTGGCACCAAAATCAACGGGACTTTCCAAAATGTCGTAACAACTCCGCCCCATTGACGCAAATGGGCGGTAG
GCGTGTACGGTGGGAGGTCTATATAAGCAGAGCTCGTTTAGTGAACCGTCAGATCTCTAGAgccgccaccATGGATTACAAAGACGATGACGATAAGC
GGGGCATGACCTTCGCCAAGATCAAATTCAGCGCCCAGATCCGGCTGGAAACCGGCCTGCACATCGGAGGATCTGATGCCTTTGCCGCTATCGG
CGCCATCGCCAGCCCTGTGATCAAGGACCCCATCACCAACCTGCCTATCATCCCCGGCTCTAGCCTGAAGGGCAAGATGAGAACACTGCTGGCCA
AGGTGTACAACGAAAAGGTGGCCGAGAAGCCTAGCGACGACAGCGACATCCTGAGCAGACTGTTCGGAAATAGCAAGGATAAGCGGTTCAAGAT
GGGCAGACTGATCTTCCGGGACGCCTTCCTGAGCAACGCCGACGAGCTGGATTCTCTGGGCGTGCGGAGCTACACCGAGGTGAAGTTCGAGAAC
ACCATCGATAGAATCACCGCCGAGGCCAATCCTAGACAGATCGAGAGAGCCATTCGGAACTCAACATTCGACTTCGAGCTGATCTACGAGATCACT
GATGAGAATGAGAACCAGGTCGAGGAAGATTTCAAGGTGATCAGAGACGGCCTGAAGCTGCTGGAACTGGACTACCTGGGCGGAAGCGGCTCCA
GAGGCTACGGCAAAGTGGCTTTTGAGAACCTGAAAGCCACCACAGTGTTCGGCAACTACGACGTGAAAACCCTGAACGAGCTGCTGACCGCCGAA
GTGggtagcaaaggagaagaacttttcactggagttgtcccaattcttgttgaattagatggtgatgttaatgggcacaaattttctgtccgtggagagggtgaaggtgatgctacaaacggaaaactcacccttaaattt
atttgcactactggaaaactacctgttccgtggccaacacttgtcactactctgacctatggtgttcaatgcttttcccgttatccggatcacatgaaacggcatgactttttcaagagtgccatgcccgaaggttatgtacagg
aacgcactatatctttcaaagatgacgggacctacaagacgcgtgctgaagtcaagtttgaaggtgatacccttgttaatcgtatcgagttaaagggtattgattttaaagaagatggaaacattcttggacacaaactcg
agtacaactttaactcacacaatgtatacatcacggcagacaaacaaaagaatggaatcaaagctaacttcaaaattcgccacaacgttgaagatggttccgttcaactagcagaccattatcaacaaaatactccaa
ttggcgatggccctgtccttttaccagacaaccattacctgtcgacacaatctgtcctttcgaaagatcccaacgaaaagcgtgaccacatggtccttcttgagtttgtaactgctgctgggattacacatggcatggatgag
ctctacaaaggtTCTaaaggagaagaacttttcactggagttgtcccaattcttgttgaattagatggtgatgttaatgggcacaaattttctgtccgtggagagggtgaaggtgatgctacaaacggaaaactcaccctt
aaatttatttgcactactggaaaactacctgttccgtggccaacacttgtcactactctgacctatggtgttcaatgcttttcccgttatccggatcacatgaaacggcatgactttttcaagagtgccatgcccgaaggttatgt
acaggaacgcactatatctttcaaagatgacgggacctacaagacgcgtgctgaagtcaagtttgaaggtgatacccttgttaatcgtatcgagttaaagggtattgattttaaagaagatggaaacattcttggacacaa
actcgagtacaactttaactcacacaatgtatacatcacggcagacaaacaaaagaatggaatcaaagctaacttcaaaattcgccacaacgttgaagatggttccgttcaactagcagaccattatcaacaaaatac
tccaattggcgatggccctgtccttttaccagacaaccattacctgtcgacacaatctgtcctttcgaaagatcccaacgaaaagcgtgaccacatggtccttcttgagtttgtaactgctgctgggattacacatggcatgg
atgagctctacaaaggttaacggcaataaaaagacagaataaaacgcacggtgttgggtcgtttgttcTTGCGAGAATGGACTAGTAGCAAACTGTGATGCGGTTTTGGCAGTACAT
CAATGGGCGTGGATAGCGGTTTGACTCACGGGGATTTCCAAGTCTCCACCCCATTGACGTCAATGGGAGTTTGTTTTGGCACCAAAATCAACGGG
ACTTTCCAAAATGTCGTAACAACTCCGCCCCATTGACGCAAATGGGCGGTAGGCGTGTACGGTGGGAGGTCTATATAAGCAGAGCTCGTTTAGTG
AACCGTCAGATCTCTAGAgccgccaccATGGATTACAAAGACGATGACGATAAGCGGGGCATGACTTACAAGCTCTACATTATGACCTTTCAAAACGCC
CACTTCGGTTCCGGCACTCTGGACTCATCGAAGCTGACCTTCTCCGCGGATAGAATCTTCTCGGCACTCGTGCTCGAGGCTCTGAAGATGGGAAA
GCTCGACGCCTTCTTGGCCGAGGCCAACCAGGATAAGTTCACTCTGACCGACGCGTTCCCATTCCAATTCGGTCCTTTCCTGCCGAAACCGATTG
GTTACCCCAAGCACGACCAGATCGACCAGTCTGTGGACGTGAAGGAAGTCCGCCGCCAAGCGAAGCTGTCCAAAAAGCTCCAGTTCCTGGCTCT
GGAAAACGTCGACGACTACCTGAACGGAGAGCTGTTTGAGAATGAGGAACACGCCGTGATCGACACAGTGACCAAGAACCAGCCCCATAAAGATG
ATAATCTGTACCAAGTGGCCACCACTCGGTTCTCGAACGACACCTCCCTTTACGTGATCGCCAACGAATCCGATCTGCTGAACGAACTGATGAGCA
GCCTTCAGTACTCCGGGCTGGGCGGCAAAAGGTCCTCAGGATTCGGCAGATTTGAGCTGGACATCCAGAACATTCCCTTGGAACTGTCCGACCGG
CTGACGAAGAACCACAGCGACAAGGTCATGTCACTTACCACCGCCCTCCCGGTGGACGCTGATCTCGAGGAAGCGATGGAAGATGGCCATTACCT
GTTGACCAAGTCGTCCGGATTCGCATTCTCCCACGCCACCAACGAAAACTATCGGAAGCAGGACCTGTACAAGTTCGCCTCCGGGAGCACCTTCA
GCAAGACTTTCGAGGGACAGATCGTGGACGTGCGCCCTCTCGATTTCCCTCACGCCGTGCTGAACTACGCCAAGCCGCTGTTCTTTAAGCTCGAA
GTCTAAcggcaataaaaagacagaataaaacgcacggtgttgggtcgtttgttcGCACATTCAAAAACAGGCAATTGGACAAGCGTGATGCGGTTTTGGCAGTACATCAAT
GGGCGTGGATAGCGGTTTGACTCACGGGGATTTCCAAGTCTCCACCCCATTGACGTCAATGGGAGTTTGTTTTGGCACCAAAATCAACGGGACTTT
CCAAAATGTCGTAACAACTCCGCCCCATTGACGCAAATGGGCGGTAGGCGTGTACGGTGGGAGGTCTATATAAGCAGAGCTCGTTTAGTGAACCG
TCAGATCTCTAGAgccgccaccATGGATTACAAAGACGATGACGATAAGCGGGGCATGAAAAATGACTACCGGACCTTCAAGCTGAGCCTGCTGACCC
TGGCTCCTATCCACATCGGCAACGGCGAGAAGTACACCAGCAGAGAATTCATCTACGAGAACAAGAAGTTCTACTTCCCCGACATGGGCAAGTTCT

ACAACAAGATGGTGGAAAAGAGACTGGCCGAGAAGTTCGAGGCCTTCCTGATCCAGACCAGACCCAACGCCAGAAACAACCGGCTGATTTCTTTT
CTGAACGACAACAGAATCGCCGAAAGATCTTTTGGCGGCTACAGCATCAGTGAAACCGGCCTGGAATCTGATAAGAACCCTAACAGCGCCGGAGC
TATCAACGAGGTGAACAAATTCATCCGGGACGCCTTCGGAAATCCTTACATCCCAGGCAGCAGCCTGAAGGGCGCCATCCGCACCATCCTGATGA
ACACCACACCTAAGTGGAACAACGAGAACGCCGTGAACGACTTCGGCAGATTCCCAAAGGAAAACAAGAACCTGATCCCTTGGGGACCTAAGAAA
GGCAAGGAATACGACGACCTGTTCAACGCCATCAGAGTGTCCGACAGCAAGCCCTTCGACAACAAAAGCCTGATCCTCGTGCAGAAGTGGGACTA
CAGCGCCAAAACCAACAAGGCCAAGCCTCTGCCTCTGTACAGAGAGTCTATCAGCCCTCTGACCAAGATCGAGTTCGAGATAACAACAACCACTG
ATGAGGCCGGCAGACTGATCGAGGAACTGGGAAAGCGGGCCCAGGCCTTTTATAAGGACTACAAGGCCTTTTTCCTGTCTGAATTCCCTGATGAT
AAGATCCAGGCTAATCTGCAATACCCCATCTACCTGGGCGCCGGCAGCGGCGCTTGGACAAAGACCCTGTTTAAGCAGGCCGACGGCATCCTGC
AGCGGAGATACTCCAGAATGAAAACCAAGATGGTCAAGAAGGGCGTGCTGAAGCTGACAAAGGCCCCTCTGAAAACAGTGAAGATCCCCAGCGG
CAACCACAGCCTGGTGAAGAATCACGAGAGCTTCTACGAGATGGGCAAAGCCAACTTCATGATCAAGGAAATCGACAAGTGAcggcaataaaaagacaga
ataaaacgcacggtgttgggtcgtttgttcATGTGTGCCGGCCGGAGAATAAAAGTCTAAGTGATGCGGTTTTGGCAGTACATCAATGGGCGTGGATAGCGGTTTG
ACTCACGGGGATTTCCAAGTCTCCACCCCATTGACGTCAATGGGAGTTTGTTTTGGCACCAAAATCAACGGGACTTTCCAAAATGTCGTAACAACTC
CGCCCCATTGACGCAAATGGGCGGTAGGCGTGTACGGTGGGAGGTCTATATAAGCAGAGCTCGTTTAGTGAACCGTCAGATCTCTAGAgccgccacc
ATGGATTACAAAGACGATGACGATAAGCGGGGCATGAAAAAGCTCGTGTTCACCTTTAAGCGGATCGACCACCCTGCTCAGGACCTGGCCGTGAA
ATTCCACGGCTTCCTGATGGAACAGCTGGATAGCGACTACGTGGACTACCTGCACCAGCAGCAGACCAACCCCTACGCCACAAAGGTGATCCAGG
GCAAAGAGAACACCCAGTGGGTCGTGCATCTGCTGACAGACGACATCGAGGACAAGGTGTTCATGACCCTGCTGCAGATCAAGGAAGTGTCCCTG
AACGACCTGCCTAAGTTGTCTGTGGAAAAGGTGGAAATCCAGGAGCTGGGCGCTGATAAGCTGCTCGAGATCTTCAACAGCGAGGAAAACCAGAC
CTACTTCAGCATCATCTTCGAGACACCTACAGGCTTTAAAAGCCAGGGCAGCTACGTGATCTTCCCCAGCATGCGGCTGATCTTTCAGAGCCTGAT
GCAGAAGTACGGCAGACTGGTGGAAAACCAGCCTGAGATCGAGGAAGATACCCTGGACTACCTGAGCGAGCACAGCACCATCACCAATTACAGA
CTGGAAACAAGCTACTTCAGAGTGCATAGACAGAGAATCCCCGCCTTCCGGGGCAAGCTGACCTTCAAGGTGCAGGGAGCCCAGACACTGAAGG
CCTACGTGAAGATGCTGCTGACCTTCGGCGAGTACAGCGGCCTGGGCATGAAAACCAGCCTGGGAATGGGCGGCATCAAGCTGGAAGAAAGAAA
GGACTGAcggcaataaaaagacagaataaaacgcacggtgttgggtcgtttgttcGTGCGATAGAGGGATCCCGCATTGAATTATGAGCAGATTGTACTGAGAGTGCACC
GGTCCGTCTTCGATGAAGCGATTCAGAAGACTTACATGTGTCAGAGGTTTTCACCGTCATCACCGAAACGCGCGAGACGAAAGGGCCTCGTGATA
CGCCTATTTTTATAGGTTAATGTCATGATAATAATGGTTTCTTAGACGTCAGGTGGCACTTTTCGGGGAAATGTGCGCGGAACCCCTATTTGTTTATT
TTTCTAAATACATTCAAATATGTATCCGCTCATGAGACAATAACCCTGATAAATGCTTCAATAATATTGAAAAAGGAAGAGTATGAGTATTCAACATTT
CCGTGTCGCCCTTATTCCCTTTTTTGCGGCATTTTGCCTTCCTGTTTTTGCTCACCCAGAAACGCTGGTGAAAGTAAAAGATGCTGAAGATCAGTTG
GGTGCACGAGTGGGTTACATCGAACTGGATCTCAACAGCGGTAAGATCCTTGAGAGTTTTCGCCCCGAAGAACGTTTTCCAATGATGAGCACTTTT
AAAGTTCTGCTATGTGGCGCGGTATTATCCCGTATTGACGCCGGGCAAGAGCAACTCGGTCGCCGCATACACTATTCTCAGAATGACTTGGTTGAG
TACTCACCAGTCACAGAAAAGCATCTTACGGATGGCATGACAGTAAGAGAATTATGCAGTGCTGCCATAACCATGAGTGATAACACTGCGGCCAAC
TTACTTCTGACAACGATCGGAGGACCGAAGGAGCTAACCGCTTTTTTGCACAACATGGGGGATCATGTAACTCGCCTTGATCGTTGGGAACCGGA
GCTGAATGAAGCCATACCAAACGACGAGCGTGACACCACGATGCCTGTAGCAATGGCAACAACGTTGCGCAAACTATTAACTGGCGAACTACTTAC
TCTAGCTTCCCGGCAACAATTAATAGACTGGATGGAGGCGGATAAAGTTGCAGGACCACTTCTGCGCTCGGCCCTTCCGGCTGGCTGGTTTATTG
CTGATAAATCTGGAGCCGGTGAGCGTGGGTCTCGCGGTATCATTGCAGCACTGGGGCCAGATGGTAAGCCCTCCCGTATCGTAGTTATCTACACG
ACGGGGAGTCAGGCAACTATGGATGAACGAAATAGACAGATCGCTGAGATAGGTGCCTCACTGATTAAGCATTGGTAACTGTCAGACCAAGTTTAC
TCATATATACTTTAGATTGATTTAAAACTTCATTTTTAATTTAAAAGGATCTAGGTGAAGATCCTTTTTGATAATCTCATGACCAAAATCCCTTAACGTG
AGTTTTCGTTCCACTGAGCGTCAGACCCCGTAGAAAAGATCAAAGGATCTTCTTGAGATCCTTTTTTTCTGCGCGTAATCTGCTGCTTGCAAACAAA
AAAACCACCGCTACCAGCGGTGGTTTGTTTGCCGGATCAAGAGCTACCAACTCTTTTTCCGAAGGTAACTGGCTTCAGCAGAGCGCAGATACCAAA
TACTGTTCTTCTAGTGTAGCCGTAGTTAGGCCACCACTTCAAGAACTCTGTAGCACCGCCTACATACCTCGCTCTGCTAATCCTGTTACCAGTGGCT
GCTGCCAGTGGCGATAAGTCGTGTCTTACCGGGTTGGACTCAAGACGATAGTTACCGGATAAGGCGCAGCGGTCGGGCTGAACGGGGGGTTCGT
GCACACAGCCCAGCTTGGAGCGAACGACCTACACCGAACTGAGATACCTACAGCGTGAGCTATGAGAAAGCGCCACGCTTCCCGAAGGGAGAAA
GGCGGACAGGTATCCGGTAAGCGGCAGGGTCGGAACAGGAGAGCGCACGAGGGAGCTTCCAGGGGGAAACGCCTGGTATCTTTATAGTCCTGT
CGGGTTTCGCCACCTCTGACTTGAGCGTCGATTTTTGTGATGCTCGTCAGGGGGGCGGAGCCTATGGAAAAACGCCAGCAACGCGGCCTTTTTCT
TAAGC

Csm1 | Csm2 | dCsm3-2xsfGFP (red indicates inactivating mutations)

Csm4 | Csm5 | Cas6 | CMV promoter

| Plasmid | pCX137 |
|---|---|
| Description | CRISPR array plasmid for *NOTCH2* (Spacers 1-24) |

GgTCGACATTGATTATTGACTAGTTATTAATAGTAATCAATTACGGGGTCATTAGTTCATAGCCCATATATGGAGTTCCGCGTTACATAACTTACGGT
AAATGGCCCGCCTGGCTGACCGCCCAACGACCCCCGCCCATTGACGTCAATAATGACGTATGTTCCCATAGTAACGCCAATAGGGACTTTCCATT
GACGTCAATGGGTGGAgTATTTACGGTAAACTGCCCACTTGGCAGTACATCAAGTGTATCATATGCCAAGTACGCCCCCTATTGACGTCAATGACG
GTAAATGGCCCGCCTGGCATTATGCCCAGTACATGACCTTATGGGACTTTCCTACTTGGCAGTACATCTACGTATTAGTCATCGCTATTACCATGGT
CGAGGTGAGCCCCACGTTCTGCTTCACTCTCCCCATCTCCCCCCCCcTCCCCACCCCCAATTTTGTATTTATTTATTTTTTAATTATTTTGTGCAGCG
ATGGGGGCGGGGGGGGGGGGGGCGCGCGCCAGGCGGGGCGGGGCGGGGCGAGGGGCGGGGCGGGGCGAGGCGGAGAGGTGCGGCGGCA
GCCAATCAGAGCGGCGCGCTCCGAAAGTTTCCTTTTATGGCGAGGCGGCGGCGGCGGCGGCCCTATAAAAAGCGAAGCGCGCGGCGGGCGGG
AGTCGCTGCGcgcTGCCTTCGCCCCGTGCCCCGCTCCGCcGCCGCCTCGCGCCGCCCGCCCCGGCTCTGACTGACCGCGTTACTCCCACAGGTG
AGCGGGCGGGACGGCCCTTCTCCTCCGGGCTGTAATTAGCGCTTGGTTTAATGACGGCTtGTTTCTTTTCTGTGGCTGCGTGAAAGCCTTgAgGGG
CTCCGGGAGGGCCCTTTGTGCGGGGGGAGCGGCTCGGGGGGTGCGTGCGTGTGTGTGTGCGTGGGGAGCGCCGCGTGCGGCtCCGCGCTGCC
CGGCGGCTGTGAGCGCTGCGGGCGCGGCGCGGGGCTTTGTGCGCTCCGCaGTGTGCGCGAGGGGAGCGCGGCCGGGGGCGGTGCCCCGCGG
TGCGGGGGGGgCTGCGAGGGGAACAAAGGCTGCGTGCGGGGTGTGTGCGTGGGGGGGTGAGCAGGGGGTGTGGGCGCGtCGGTCGGGCTGcA
ACCCCCCCTGCACCCCCCTCCCCGAGTTGCTGAGCACGGCCCGGCTTCGGGTGCGGGGCTCCGTaCGGGGCGTGGCGCGGGGCTCGCCGTGC
CGGGCGGGGGGTGGCGGCAGGTGGGGGTGCCGGGCGGGGCGGGGCCGCCTCGGGCCGGGGAGGGCTCGGGGGAGGGGCGCGGCGGCCCC
cGGAGCGCCGGCGGCTGTCGAGGCGCGGCGAGCCGCAGCCATTGCCTTTTATGGTAATCGTGCGAGAGGGCGCAGGGACTTCCTTTGTCCCAAA
TCTGtGCGGAGCCGAAATCTGGGAGGCGCCGCCGCACCCCCTCTAGCGGGCGCGGGgCGAAGCGGTGCGGCGCCGGCAGGAAGGAAATGGGC
GGGGAGGGCCTTCGTGCGTCGCCGCGCCGCCGTCCCCTTCTCCcTCTCCAGCCTCGGGGCTGtCCGCgGGGGGACGGCTGCCTTCGGGGGGGA
CGGGGCAGGGCGGGGTTCGGCTTCTGGCGTGTGACCGGCGGCTCTAGAGCCTCTGCTAACCATGTTCATGCCTTCTTCTTTTTCCTACAGCTCCT
GGGCAACGTGCTGGTTaTTGTGCTGTCTCATCATTTTGGCAAAGAATTCgccaccgtggactcagcggcagagacgccaccccgagaaggcaaatcccactttcagatcctgctg
gacgtggtccagttcctccctgaagacatcatcattcagaccttcgaaggctggctgctgataaaagcacaacacggaaccagaatggatgagcacggttttatctcaagaagcttcacccgacagtacaaactacca
gatggtgtggaaatcaaagatttgtctgcagtcctctgtcatgatggaattttggtggtggaagtaaaggatccagttgggactaagGAGCTCatcgaatccttaagctacgaacgatataaacctaattacctcgaga
ggggacggaaacTTTGTTCCTCAGCAGCATTTACAAAAGTCAGTTATGgatataaacctaattacctcgagaggggacggaaacTACCCAGTGAAACTGACGAATTGCTT
CTCTTGCATTgatataaacctaattacctcgagaggggacggaaacTACCTCTAGAAGCTGGCTCCAGAGATTTCTTCATTTgatataaacctaattacctcgagaggggacggaaa
cTTGTATTCATCTTGCATTTCCACAAACTTGTCTTATgatataaacctaattacctcgagaggggacggaaacTTCAATAAGCATCCATCTTATTCTCCAAATAGAAGA
gatataaacctaattacctcgagaggggacggaaacTTCTCATGGATATGGCAGAAGCCTGCAGGGCTTAAAgatataaacctaattacctcgagaggggacggaaacTTTCATGG
GCCAGAATGATCAAAAGCACAAAGGTCTgatataaacctaattacctcgagaggggacggaaacTTTATATGAAGACCTGCACACAGACAAGAAAAATTAgatataaacc
taattacctcgagaggggacggaaacTTTGTGCTGAGAACCATACCAAAGTAAACCTTGTGAgatataaacctaattacctcgagaggggacggaaacTTCTCTCTCCAGGAAT
GATATATGCAGGAGAACACAgatataaacctaattacctcgagaggggacggaaacTTCCAAACCTTTTTCTGGTTTTAAAAAAGTGGGGAGgatataaacctaattacctcga
gaggggacggaaacTTTACACTGACCAGGCCCATACACAGAAAAACAACAgatataaacctaattacctcgagaggggacggaaacAAACAGGCCTAGAATGCTCAGAT
CTGGGACAAATGTgatataaacctaattacctcgagaggggacggaaacTTGGCAGGAAGTAGGGGGACCTGTGGGTGGGTAACTgatataaacctaattacctcgagagggg
acggaaacTTCTGTGGATATGAAAGGAAAGGCTCAACAGTTAAGgatataaacctaattacctcgagaggggacggaaacTTGGTGAGCAGGCCTTTAAGCAGCAAGA
TAATCAGTgatataaacctaattacctcgagaggggacggaaacTGTCACTGGGTCCCTTGGGCTAAGGAAAGTTCAGTCgatataaacctaattacctcgagaggggacggaaac
TATAAAGTCCATGTCTTCAGTGAGAACATACTGGGTgatataaacctaattacctcgagaggggacggaaacTTCTTATAGGGCTGTTTTGGATCATAGAAAACAAG
TgatataaacctaattacctcgagaggggacggaaacTAGGAAAGGAAAATGTTCTGTTGAGTTTCTACGTTAgatataaacctaattacctcgagaggggacggaaacTTCAGAGG
AAAAGAAATGGTAGCAGAAAATAGCAAAgatataaacctaattacctcgagaggggacggaaacTTCAGGTGAGGGGCAATCAGTCTGAACAATGAGCAAgatataaac
ctaattacctcgagaggggacggaaacTTAACTAAAATGCTTCTGCCCTTAAAAGAAAGTCAAgatataaacctaattacctcgagaggggacggaaacTTGCAGTGTAGCCAT
GGACCAATTATTAACTGGCCCgatataaacctaattacctcgagaggggacggaaacaccggtGCAATTACGCTTAgCTAGCAGATCTTTTTCCCTCTGCCAAAAATTA
TGGGGACATCATGAAGCCCCTTGAGCATCTGACTTCTGGCTAATAAAGGAAATTTATTTTCATTGCAATAGTGTGTTGGAATTTTTTGTGTCTCTCAC
TCGGAAGGACATATGGGAGGGCAAATCATTTAAAACATCAGAATGAGTATTTGGTTTAGAGTTTGGCAACATATGCCCATATGCTGGCTGCCATGAA
CAAAGGTTGGCTATAAAGAGGTCATCAGTATATGAAACAGCCCCCTGCTGTCCATTCCTTATTCCATAGAAAAGCCTTGACTTGAGGTTAGATTTTTT
TTATATTTTGTTTTGTGTTATTTTTTTCTTTAACATCCCTAAAATTTTCCTTACATGTTTTACTAGCCAGATTTTTCCTCCTCTCCTGACTACTCCCAGTC
ATAGCTGTCCCTCTTCTCTTATGgAGATCCCTCGACCTGCAGCCCAAGCTTGGCGTAATCATGGTCATAGCTGTTTCCTGTGTGAAATTGTTATCCG
CTCACAATTCCACACAACATACGAGCCGGAAGCATAAAGTGTAAAGCCTGGGGTGCCTAATGAGTGAGCTAACTCACATTAATTGCGTTGCGCTCA
CTGCCCGCTTTCCAGTCGGGAAACCTGTCGTGCCAGCGGATCCGCATCTCAATTAGTCAGCAACCATAGTCCCGCCCCTAACTCCGCCCATCCCG
CCCCTAACTCCGCCCAGTTCCGCCCATTCTCCGCCCCATGGCTGACTAATTTTTTTTATTTATGCAGAGGCCGAGGCCGCCTCGGCCTCTGAGCTA
TTCCAGAAGTAGTGAGGAGGCTTTTTTGGAGGCCTAGGCTTTTGCAAAAAGCTAACTTGTTTATTGCAGCTTATAATGGTTACAAATAAAGCAATAG
CATCACAAATTTCACAAATAAAGCATTTTTTTCACTGCATTCTAGTTGTGGTTTGTCCAAACTCATCAATGTATCTTATCATGTCTGGATCCGCTGCAT
TAATGAATCGGCCAACGCGCGGGGAGAGGCGGTTTGCGTATTGGGCGCTCTTCCGCTTCCTCGCTCACTGACTCGCTGCGCTCGGTCGTTCGGC
TGCGGCGAGCGGTATCAGCTCACTCAAAGGCGGTAATACGGTTATCCACAGAATCAGGGGATAACGCAGGAAAGAACATGTGAGCAAAAGGCCA
GCAAAAGGCCAGGAACCGTAAAAAGGCCGCGTTGCTGGCGTTTTTCCATAGGCTCCGCCCCCCTGACGAGCATCACAAAAATCGACGCTCAAGTC
AGAGGTGGCGAAACCCGACAGGACTATAAAGATACCAGGCGTTTCCCCCTGGAAGCTCCCTCGTGCGCTCTCCTGTTCCGACCCTGCCGCTTACC
GGATACCTGTCCGCCTTTCTCCCTTCGGGAAGCGTGGCGCTTTCTCAtaGCTCACGCTGTAGGTATCTCAGTTCGGTGTAGGTCGTTCGCTCCAAG
CTGGGCTGTGTGCACGAACCCCCCGTTCAGCCCGACCGCTGCGCCTTATCCGGTAACTATCGTCTTGAGTCCAACCCGGTAAGACACGACTTATC
GCCACTGGCAGCAGCCACTGGTAACAGGATTAGCAGAGCGAGGTATGTAGGCGGTGCTACAGAGTTCTTGAAGTGGTGGCCTAACTACGGCTAC
ACTAGAAGaACAGTATTTGGTATCTGCGCTCTGCTGAAGCCAGTTACCTTCGGAAAAAGAGTTGGTAGCTCTTGATCCGGCAAACAAACCACCGCT
GGTAGCGGTGGTTTTTTTGTTTGCAAGCAGCAGATTACGCGCAGAAAAAAAGGATCTCAAGAAGATCCTTTGATCTTTTCTACGGGGTCTGACGCT
CAGTGGAACGAAAACTCACGTTAAGGGATTTTGGTCATGAGATTATCAAAAAGGATCTTCACCTAGATCCTTTTAAATTAAAAATGAAGTTTTAAATC
AATCTAAAGTATATATGAGTAAACTTGGTCTGACAGTTACCAATGCTTAATCAGTGAGGCACCTATCTCAGCGATCTGTCTATTTCGTTCATCCATAG
TTGCCTGACTCCCCGTCGTGTAGATAACTACGATACGGGAGGGCTTACCATCTGGCCCCAGTGCTGCAATGATACCGCGAGACCCACGCTCACCG
GCTCCAGATTTATCAGCAATAAACCAGCCAGCCGGAAGGGCCGAGCGCAGAAGTGGTCCTGCAACTTTATCCGCCTCCATCCAGTCTATTAATTGT
TGCCGGGAAGCTAGAGTAAGTAGTTCGCCAGTTAATAGTTTGCGCAACGTTGTTGCCATTGCTACAGGCATCGTGGTGTCACGCTCGTCGTTTGGT
ATGGCTTCATTCAGCTCCGGTTCCCAACGATCAAGGCGAGTTACATGATCCCCCATGTTGTGCAAAAAAGCGGTTAGCTCCTTCGGTCCTCCGATC
GTTGTCAGAAGTAAGTTGGCCGCAGTGTTATCACTCATGGTTATGGCAGCACTGCATAATTCTCTTACTGTCATGCCATCCGTAAGATGCTTTTCTG
TGACTGGTGAGTACTCAACCAAGTCATTCTGAGAATAGTGTATGCGGCGACCGAGTTGCTCTTGCCCGGCGTCAATACGGGATAATACCGCGCCA
CATAGCAGAACTTTAAAAGTGCTCATCATTGGAAAACGTTCTTCGGGGCGAAAACTCTCAAGGATCTTACCGCTGTTGAGATCCAGTTCGATGTAAC
CCACTCGTGCACCCAACTGATCTTCAGCATCTTTTACTTTCACCAGCGTTTCTGGGTGAGCAAAAACAGGAAGGCAAAATGCCGCAAAAAAGGGAA
TAAGGGCGACACGGAAATGTTGAATACTCATACTCTTCCTTTTTCAATATTATTGAAGCATTTATCAGGGTTATTGTCTCATGAGCGGATACATATTT
GAATGTATTTAGAAAAATAAACAAATAGGGGTTCCGCGCACATTTCCCCGAAAAGTGCCACCTG

CAG promoter | HSPB3 export signal | *Sth*Csm direct repeat sequence

| Plasmid | pCX138 |
|---|---|
| Description | CRISPR array plasmid for *NOTCH2* (Spacers 25-48) |

ggTCGACATTGATTATTGACTAGTTATTAATAGTAATCAATTACGGGGTCATTAGTTCATAGCCCATATATGGAGTTCCGCGTTACATAACTTACGGTA
AATGGCCCGCCTGGCTGACCGCCCAACGACCCCCGCCCATTGACGTCAATAATGACGTATGTTCCCATAGTAACGCCAATAGGGACTTTCCATTG
ACGTCAATGGGTGGAgTATTTACGGTAAACTGCCCACTTGGCAGTACATCAAGTGTATCATATGCCAAGTACGCCCCCTATTGACGTCAATGACGG
TAAATGGCCCGCCTGGCATTATGCCCAGTACATGACCTTATGGGACTTTCCTACTTGGCAGTACATCTACGTATTAGTCATCGCTATTACCATGGTC
GAGGTGAGCCCCACGTTCTGCTTCACTCTCCCCATCTCCCCCCCCcTCCCCACCCCCAATTTTGTATTTATTTATTTTTTAATTATTTTGTGCAGCGA
TGGGGGCGGGGGGGGGGGGGGCGCGCGCCAGGCGGGGCGGGGCGGGGCGAGGGGCGGGGCGGGGCGAGGCGGAGAGGTGCGGCGGCAG
CCAATCAGAGCGGCGCGCTCCGAAAGTTTCCTTTTATGGCGAGGCGGCGGCGGCGGCGGCCCTATAAAAAGCGAAGCGCGCGGCGGGCGGGA
GTCGCTGCGcgcTGCCTTCGCCCCGTGCCCCGCTCCGCcGCCGCCTCGCGCCGCCCGCCCCGGCTCTGACTGACCGCGTTACTCCCACAGGTGA
GCGGGCGGGACGGCCCTTCTCCTCCGGGCTGTAATTAGCGCTTGGTTTAATGACGGCTtGTTTCTTTTCTGTGGCTGCGTGAAAGCCTTgAgGGGC
TCCGGGAGGGCCCTTTGTGCGGGGGGAGCGGCTCGGGGGGTGCGTGCGTGTGTGTGTGCGTGGGGAGCGCCGCGTGCGGCtCCGCGCTGCCC
GGCGGCTGTGAGCGCTGCGGGCGCGGCGCGGGGCTTTGTGCGCTCCGCaGTGTGCGCGAGGGGAGCGCGGCCGGGGGCGGTGCCCCGCGGT
GCGGGGGGGgCTGCGAGGGGAACAAAGGCTGCGTGCGGGGTGTGTGCGTGGGGGGGTGAGCAGGGGGTGTGGGCGCGtCGGTCGGGCTGcAA
CCCCCCCTGCACCCCCCTCCCCGAGTTGCTGAGCACGGCCCGGCTTCGGGTGCGGGGCTCCGTaCGGGGCGTGGCGCGGGGCTCGCCGTGCC
GGGCGGGGGGTGGCGGCAGGTGGGGGTGCCGGGCGGGGCGGGGCCGCCTCGGGCCGGGGAGGGCTCGGGGGAGGGGCGCGGCGGCCCCc
GGAGCGCCGGCGGCTGTCGAGGCGCGGCGAGCCGCAGCCATTGCCTTTTATGGTAATCGTGCGAGAGGGCGCAGGGACTTCCTTTGTCCCAAAT
CTGtGCGGAGCCGAAATCTGGGAGGCGCCGCCGCACCCCCTCTAGCGGGCGCGGGgCGAAGCGGTGCGGCGCCGGCAGGAAGGAAATGGGCG
GGGAGGGCCTTCGTGCGTCGCCGCGCCGCCGTCCCCTTCTCCcTCTCCAGCCTCGGGGCTGtCCGCgGGGGGACGGCTGCCTTCGGGGGGGAC
GGGGCAGGGCGGGGTTCGGCTTCTGGCGTGTGACCGGCGGCTCTAGAGCCTCTGCTAACCATGTTCATGCCTTCTTCTTTTTCCTACAGCTCCTG
GGCAACGTGCTGGTTaTTGTGCTGTCTCATCATTTTGGCAAAGAATTCgccaccgtggactcagcggcagagacgccaccccgagaaggcaaatcccactttcagatcctgctgga
cgtggtccagttcctccctgaagacatcatcattcagaccttcgaaggctggctgctgataaaagcacaacacggaaccagaatggatgagcacggttttatctcaagaagcttcacccgacagtacaaactaccaga
tggtgtggaaatcaaagatttgtctgcagtcctctgtcatgatggaattttggtggtggaagtaaaggatccagttgggactaagGAGCTCATCGAATCCTTAAGCTAcgaacgatataaacctaattacct
cgagaggggacggaaacAAAGAGTCGGGAATTCACCTGTTAATATCTACAAAAgatataaacctaattacctcgagaggggacggaaacATAGGACTGAAAAAACCATTTG
TAAACTATTATGCCgatataaacctaattacctcgagaggggacggaaacAAATCAGGTAAGTGGGAAGCACTGATGCATACTTGCgatataaacctaattacctcgagaggga
cggaaacAAATGGGTCCAAGGGCAGAGAGTCACCATTTAGAATgatataaacctaattacctcgagaggggacggaaacAAAGCAGAGAATGAACAAACTTAATCTGC
TTATCTCgatataaacctaattacctcgagaggggacggaaacAGGGCAGAAAGGGGCAGGTACGCTGTGGTCCACAGAgatataaacctaattacctcgagaggggacggaaac
AAAAGGGAGGATATAAAATATCCAGTGGCTGGATCAgatataaacctaattacctcgagaggggacggaaacATGGCATAAAAGATGTGTCTCATAAAAACTGAGTC
TgatataaacctaattacctcgagaggggacggaaacAAAGGCAGTGTTGTCCTCCTCATCACTGAAACCTGTgatataaacctaattacctcgagaggggacggaaacAAACCCAA
AGGATCCAAATTAAAGTAGTCCAAGAAAgatataaacctaattacctcgagaggggacggaaacTTACCTTCCCTTTTATTAGATACCACACAATTCAAGgatataaacct
aattacctcgagaggggacggaaacAGAAGAGGAGTCAAAGAAAGAAAGCATTTATAACAAgatataaacctaattacctcgagaggggacggaaacACCTCACATAAGAAAA
TGATGCTTAAAACAAAACAGgatataaacctaattacctcgagaggggacggaaacAAAACATTACACCTTTGGTTCTTTATTATGCAAAAAgatataaacctaattacctcgag
aggggacggaaacTTTGAGCATCACAGCCAATTGCTTATACTAAAATATgatataaacctaattacctcgagaggggacggaaacAAATGTGTCCTCCCAAATAGTCATC
ATACATTCAATgatataaacctaattacctcgagaggggacggaaacAAAGTGTGCATTTTCCTTACTACGTTTAGTCAGGAAgatataaacctaattacctcgagaggggacggaa
acATATCACTTTTAAGAGAAATGTACACAAGGAAGTAAgatataaacctaattacctcgagaggggacggaaacTTTTGTGATAAAAATGCTTTCATATAAATTTCATC
TgatataaacctaattacctcgagaggggacggaaacTTTGTGGGATTCAGAAAGAAAAGAAAATTTGCTCTCgatataaacctaattacctcgagaggggacggaaacTGCCAGGC
CATGGATGCAGTATTGGAAAATAAAAAAgatataaacctaattacctcgagaggggacggaaacTTTCCCCAGGATCATTTATTTATGATCTAATTAAAGgatataaaccta
attacctcgagaggggacggaaacTTCCCAATTCTTGCTATAGCAACTACTTCGCATTTCgatataaacctaattacctcgagaggggacggaaacTTTGTCACCAGCTATGGC
TAGTGCACAGAAGAGTGCgatataaacctaattacctcgagaggggacggaaacAGGTTAACACTGCCCTTGAAGCTAGCAGATCTTTTTCCCTCTGCCAAAAATTA
TGGGGACATCATGAAGCCCCTTGAGCATCTGACTTCTGGCTAATAAAGGAAATTTATTTTCATTGCAATAGTGTGTTGGAATTTTTTGTGTCTCTCAC
TCGGAAGGACATATGGGAGGGCAAATCATTTAAAACATCAGAATGAGTATTTGGTTTAGAGTTTGGCAACATATGCCCATATGCTGGCTGCCATGAA
CAAAGGTTGGCTATAAAGAGGTCATCAGTATATGAAACAGCCCCCTGCTGTCCATTCCTTATTCCATAGAAAAGCCTTGACTTGAGGTTAGATTTTTT
TTATATTTTGTTTTGTGTTATTTTTTTCTTTAACATCCCTAAAATTTTCCTTACATGTTTTACTAGCCAGATTTTTCCTCCTCTCCTGACTACTCCCAGTC
ATAGCTGTCCCTCTTCTCTTATGgAGATCCCTCGACCTGCAGCCCAAGCTTGGCGTAATCATGGTCATAGCTGTTTCCTGTGTGAAATTGTTATCCG
CTCACAATTCCACACAACATACGAGCCGGAAGCATAAAGTGTAAAGCCTGGGGTGCCTAATGAGTGAGCTAACTCACATTAATTGCGTTGCGCTCA
CTGCCCGCTTTCCAGTCGGGAAACCTGTCGTGCCAGCGGATCCGCATCTCAATTAGTCAGCAACCATAGTCCCGCCCCTAACTCCGCCCATCCCG
CCCCTAACTCCGCCCAGTTCCGCCCATTCTCCGCCCCATGGCTGACTAATTTTTTTTATTTATGCAGAGGCCGAGGCCGCCTCGGCCTCTGAGCTA
TTCCAGAAGTAGTGAGGAGGCTTTTTTGGAGGCCTAGGCTTTTGCAAAAAGCTAACTTGTTTATTGCAGCTTATAATGGTTACAAATAAAGCAATAG
CATCACAAATTTCACAAATAAAGCATTTTTTTCACTGCATTCTAGTTGTGGTTTGTCCAAACTCATCAATGTATCTTATCATGTCTGGATCCGCTGCAT
TAATGAATCGGCCAACGCGCGGGGAGAGGCGGTTTGCGTATTGGGCGCTCTTCCGCTTCCTCGCTCACTGACTCGCTGCGCTCGGTCGTTCGGC
TGCGGCGAGCGGTATCAGCTCACTCAAAGGCGGTAATACGGTTATCCACAGAATCAGGGGATAACGCAGGAAAGAACATGTGAGCAAAAGGCCA
GCAAAAGGCCAGGAACCGTAAAAAGGCCGCGTTGCTGGCGTTTTTCCATAGGCTCCGCCCCCCTGACGAGCATCACAAAAATCGACGCTCAAGTC
AGAGGTGGCGAAACCCGACAGGACTATAAAGATACCAGGCGTTTCCCCCTGGAAGCTCCCTCGTGCGCTCTCCTGTTCCGACCCTGCCGCTTACC
GGATACCTGTCCGCCTTTCTCCCTTCGGGAAGCGTGGCGCTTTCTCAtaGCTCACGCTGTAGGTATCTCAGTTCGGTGTAGGTCGTTCGCTCCAAG
CTGGGCTGTGTGCACGAACCCCCCGTTCAGCCCGACCGCTGCGCCTTATCCGGTAACTATCGTCTTGAGTCCAACCCGGTAAGACACGACTTATC
GCCACTGGCAGCAGCCACTGGTAACAGGATTAGCAGAGCGAGGTATGTAGGCGGTGCTACAGAGTTCTTGAAGTGGTGGCCTAACTACGGCTAC
ACTAGAAGaACAGTATTTGGTATCTGCGCTCTGCTGAAGCCAGTTACCTTCGGAAAAAGAGTTGGTAGCTCTTGATCCGGCAAACAAACCACCGCT
GGTAGCGGTGGTTTTTTTGTTTGCAAGCAGCAGATTACGCGCAGAAAAAAAGGATCTCAAGAAGATCCTTTGATCTTTTCTACGGGGTCTGACGCT
CAGTGGAACGAAAACTCACGTTAAGGGATTTTGGTCATGAGATTATCAAAAAGGATCTTCACCTAGATCCTTTTAAATTAAAAATGAAGTTTTAAATC
AATCTAAAGTATATATGAGTAAACTTGGTCTGACAGTTACCAATGCTTAATCAGTGAGGCACCTATCTCAGCGATCTGTCTATTTCGTTCATCCATAG
TTGCCTGACTCCCCGTCGTGTAGATAACTACGATACGGGAGGGCTTACCATCTGGCCCCAGTGCTGCAATGATACCGCGAGACCCACGCTCACCG
GCTCCAGATTTATCAGCAATAAACCAGCCAGCCGGAAGGGCCGAGCGCAGAAGTGGTCCTGCAACTTTATCCGCCTCCATCCAGTCTATTAATTGT
TGCCGGGAAGCTAGAGTAAGTAGTTCGCCAGTTAATAGTTTGCGCAACGTTGTTGCCATTGCTACAGGCATCGTGGTGTCACGCTCGTCGTTTGGT
ATGGCTTCATTCAGCTCCGGTTCCCAACGATCAAGGCGAGTTACATGATCCCCCATGTTGTGCAAAAAAGCGGTTAGCTCCTTCGGTCCTCCGATC
GTTGTCAGAAGTAAGTTGGCCGCAGTGTTATCACTCATGGTTATGGCAGCACTGCATAATTCTCTTACTGTCATGCCATCCGTAAGATGCTTTTCTG
TGACTGGTGAGTACTCAACCAAGTCATTCTGAGAATAGTGTATGCGGCGACCGAGTTGCTCTTGCCCGGCGTCAATACGGGATAATACCGCGCCA
CATAGCAGAACTTTAAAAGTGCTCATCATTGGAAAACGTTCTTCGGGGCGAAAACTCTCAAGGATCTTACCGCTGTTGAGATCCAGTTCGATGTAAC
CCACTCGTGCACCCAACTGATCTTCAGCATCTTTTACTTTCACCAGCGTTTCTGGGTGAGCAAAAACAGGAAGGCAAAATGCCGCAAAAAAGGGAA
TAAGGGCGACACGGAAATGTTGAATACTCATACTCTTCCTTTTTCAATATTATTGAAGCATTTATCAGGGTTATTGTCTCATGAGCGGATACATATTT
GAATGTATTTAGAAAAATAAACAAATAGGGGTTCCGCGCACATTTCCCCGAAAAGTGCCACCTG

CAG promoter — HSPB3 export signal — *Sth*Csm direct repeat sequence

| | |
|---|---|
| Plasmid | pCX459 |
| Description | CRISPR array plasmid for *MAP1B* (Spacers 1-24) |

GgTCGACATTGATTATTGACTAGTTATTAATAGTAATCAATTACGGGGTCATTAGTTCATAGCCCATATATGGAGTTCCGCGTTACATAACTTACGGTAAAT
GGCCCGCCTGGCTGACCGCCCAACGACCCCCGCCCATTGACGTCAATAATGACGTATGTTCCCATAGTAACGCCAATAGGGACTTTCCATTGACGT
CAATGGGTGGAgTATTTACGGTAAACTGCCCACTTGGCAGTACATCAAGTGTATCATATGCCAAGTACGCCCCCTATTGACGTCAATGACGGTAAATG
GCCCGCCTGGCATTATGCCCAGTACATGACCTTATGGGACTTTCCTACTTGGCAGTACATCTACGTATTAGTCATCGCTATTACCATGGTCGAGGTGA
GCCCCACGTTCTGCTTCACTCTCCCCATCTCCCCCCCCcTCCCCACCCCCAATTTTGTATTTATTTATTTTTTAATTATTTTGTGCAGCGATGGGGGCG
GGGGGGGGGGGGGCGCGCGCCAGGCGGGGCGGGGCGGGGCGAGGGGCGGGGCGGGGCGAGGCGGAGAGGTGCGGCGGCAGCCAATCAGA
GCGGCGCGCTCCGAAAGTTTCCTTTTATGGCGAGGCGGCGGCGGCGGCGGCCCTATAAAAAGCGAAGCGCGCGGCGGGCGGGAGTCGCTGCGc
gcTGCCTTCGCCCCGTGCCCCGCTCCGCcGCCGCCTCGCGCCGCCCGCCCCGGCTCTGACTGACCGCGTTACTCCCACAGGTGAGCGGGCGGG
ACGGCCCTTCTCCTCCGGGCTGTAATTAGCGCTTGGTTTAATGACGGCTtGTTTCTTTTCTGTGGCTGCGTGAAAGCCTTgAgGGGCTCCGGGAGGG
CCCTTTGTGCGGGGGGAGCGGCTCGGGGGGTGCGTGCGTGTGTGTGTGCGTGGGGAGCGCCGCGTGCGGCtCCGCGCTGCCCGGCGGCTGTG
AGCGCTGCGGGCGCGGCGCGGGGCTTTGTGCGCTCCGCaGTGTGCGCGAGGGGAGCGCGGCCGGGGGCGGTGCCCCGCGGTGCGGGGGGG
gCTGCGAGGGGAACAAAGGCTGCGTGCGGGGTGTGTGCGTGGGGGGGTGAGCAGGGGGTGTGGGCGCGtCGGTCGGGCTGcAACCCCCCCTG
CACCCCCCTCCCCGAGTTGCTGAGCACGGCCCGGCTTCGGGTGCGGGGCTCCGTaCGGGGCGTGGCGCGGGGCTCGCCGTGCCGGGCGGGG
GGTGGCGGCAGGTGGGGGTGCCGGGCGGGGCGGGGCCGCCTCGGGCCGGGGAGGGCTCGGGGGAGGGGCGCGGCGGCCCCcGGAGCGCC
GGCGGCTGTCGAGGCGCGGCGAGCCGCAGCCATTGCCTTTTATGGTAATCGTGCGAGAGGGCGCAGGGACTTCCTTTGTCCCAAATCTGtGCGGA
GCCGAAATCTGGGAGGCGCCGCCGCACCCCCTCTAGCGGGCGCGGGgCGAAGCGGTGCGGCGCCGGCAGGAAGGAAATGGGCGGGGAGGGC
CTTCGTGCGTCGCCGCGCCGCCGTCCCCTTCTCCcTCTCCAGCCTCGGGGCTGtCCGCgGGGGGACGGCTGCCTTCGGGGGGGACGGGGCAGG
GCGGGGTTCGGCTTCTGGCGTGTGACCGGCGGCTCTAGAGCCTCTGCTAACCATGTTCATGCCTTCTTCTTTTTCCTACAGCTCCTGGGCAACGTG
CTGGTTaTTGTGCTGTCTCATCATTTTGGCAAAGAATTCgccaccgtggactcagcggcagagacgccaccccgagaaggcaaatcccactttcagatcctgctggacgtggtccagttcct
ccctgaagacatcatcattcagaccttcgaaggctggctgctgataaaagcacaacacggaaccagaatggatgagcacggttttatctcaagaagcttcacccgacagtacaaactaccagatggtgtggaaatca
aagatttgtctgcagtcctctgtcatgatggaattttggtggtggaagtaaaggatccagttgggactaagGAGCTCatcgaatccttaagctacgaacgatataaacctaattacctcgagaggggacggaaacTT
TCTGGAAACAAAGTTCAGATCCTGTGGTGTgatataaacctaattacctcgagaggggacggaaacCCTTAGCAATCAGCAAAAACTGAGAAATTTAGgatataaacctaat
tacctcgagaggggacggaaacCAGGTAATTGTTCAGCGACTTAACTAGATGAAgatataaacctaattacctcgagaggggacggaaacGAGATGAACTGGGGTAGGTAATT
TTGCAGGAAgatataaacctaattacctcgagaggggacggaaacGACAAATGCCTCTACCTAGCAGAAGTCAGTTGgatataaacctaattacctcgagaggggacggaaacTCTC
TCCTGCGTTATCTTTGTGCTCTCACTCTgatataaacctaattacctcgagaggggacggaaacTCACCAGAACTCTCTGTCATTACATCTCCTTCgatataaacctaattacct
cgagaggggacggaaacACTGACAAAAATGGAAAACTTTGCCTATGTAGgatataaacctaattacctcgagaggggacggaaacCCTAATTGTTGGTTTATTTTCTCCTCC
TTACCgatataaacctaattacctcgagaggggacggaaacGTTGTTCTTTTTACTTTCTTCTAGCCAACTTGgatataaacctaattacctcgagaggggacggaaacGTGTGCAGC
CTTCAAACTGAAGGAGCTTAATTgatataaacctaattacctcgagaggggacggaaacGTCTGAGAGTTCTGAATGGGGCCCAAACCACTgatataaacctaattacctcgag
aggggacggaaacCAACTGCTTTGGTATTTGCTTCAGTTAATTGGgatataaacctaattacctcgagaggggacggaaacTCAGAGTGGACTAAGTGGGTGGACAAATG
CTGgatataaacctaattacctcgagaggggacggaaacAAGTGCATCAAGTATCTTTGTACATTCAAGTCgatataaacctaattacctcgagaggggacggaaacCATGACTCAG
CATCAAACTGTTGGACCAATCAgatataaacctaattacctcgagaggggacggaaacCGTGTTCCTTTAAGTATCTATAATCCTATATGgatataaacctaattacctcgagaggg
gacggaaacAAGCTGTGAAAGGCACAACTTGTTTGACTGAGgatataaacctaattacctcgagaggggacggaaacAAGATTCACTGTTTTCCCAGTTCTGATCATAG
gatataaacctaattacctcgagaggggacggaaacAAGCCAATGTAAGACAAAGGAAGGTAAATACTgatataaacctaattacctcgagaggggacggaaacTAAATGAAAACATT
TGTTAAAAAGTTCATCTAgatataaacctaattacctcgagaggggacggaaacTCTTCTGTACACTTGTTCCCATAATTATGTTGgatataaacctaattacctcgagaggggacgg
aaacCATTATTCAATTTTTGTCTTACGAAACACTTAgatataaacctaattacctcgagaggggacggaaacGTTATGGGTTTTTAATAAATATCATTACTGCAgatataaacct
aattacctcgagaggggacggaaacTCTCACGTACGACTGATGCAGCTAGCAGATCTTTTTCCCTCTGCCAAAAATTATGGGGACATCATGAAGCCCCTTGAGC
ATCTGACTTCTGGCTAATAAAGGAAATTTATTTTCATTGCAATAGTGTGTTGGAATTTTTTGTGTCTCTCACTCGGAAGGACATATGGGAGGGCAAATC
ATTTAAAACATCAGAATGAGTATTTGGTTTAGAGTTTGGCAACATATGCCCATATGCTGGCTGCCATGAACAAAGGTTGGCTATAAAGAGGTCATCAGT
ATATGAAACAGCCCCCTGCTGTCCATTCCTTATTCCATAGAAAAGCCTTGACTTGAGGTTAGATTTTTTTTATATTTTGTTTTGTGTTATTTTTTTCTTTAA
CATCCCTAAAATTTTCCTTACATGTTTTACTAGCCAGATTTTTCCTCCTCTCCTGACTACTCCCAGTCATAGCTGTCCCTCTTCTCTTATGgAGATCCCT
CGACCTGCAGCCCAAGCTTGGCGTAATCATGGTCATAGCTGTTTCCTGTGTGAAATTGTTATCCGCTCACAATTCCACACAACATACGAGCCGGAAG
CATAAAGTGTAAAGCCTGGGGTGCCTAATGAGTGAGCTAACTCACATTAATTGCGTTGCGCTCACTGCCCGCTTTCCAGTCGGGAAACCTGTCGTGC
CAGCGGATCCGCATCTCAATTAGTCAGCAACCATAGTCCCGCCCCTAACTCCGCCCATCCCGCCCCTAACTCCGCCCAGTTCCGCCCATTCTCCGC
CCCATGGCTGACTAATTTTTTTTATTTATGCAGAGGCCGAGGCCGCCTCGGCCTCTGAGCTATTCCAGAAGTAGTGAGGAGGCTTTTTTGGAGGCCT
AGGCTTTTGCAAAAAGCTAACTTGTTTATTGCAGCTTATAATGGTTACAAATAAAGCAATAGCATCACAAATTTCACAAATAAAGCATTTTTTTCACTGCA
TTCTAGTTGTGGTTTGTCCAAACTCATCAATGTATCTTATCATGTCTGGATCCGCTGCATTAATGAATCGGCCAACGCGCGGGGAGAGGCGGTTTGC
GTATTGGGCGCTCTTCCGCTTCCTCGCTCACTGACTCGCTGCGCTCGGTCGTTCGGCTGCGGCGAGCGGTATCAGCTCACTCAAAGGCGGTAATA
CGGTTATCCACAGAATCAGGGGATAACGCAGGAAAGAACATGTGAGCAAAAGGCCAGCAAAAGGCCAGGAACCGTAAAAAGGCCGCGTTGCTGGC
GTTTTTCCATAGGCTCCGCCCCCCTGACGAGCATCACAAAAATCGACGCTCAAGTCAGAGGTGGCGAAACCCGACAGGACTATAAAGATACCAGGC
GTTTCCCCCTGGAAGCTCCCTCGTGCGCTCTCCTGTTCCGACCCTGCCGCTTACCGGATACCTGTCCGCCTTTCTCCCTTCGGGAAGCGTGGCGC
TTTCTCAtaGCTCACGCTGTAGGTATCTCAGTTCGGTGTAGGTCGTTCGCTCCAAGCTGGGCTGTGTGCACGAACCCCCCGTTCAGCCCGACCGCT
GCGCCTTATCCGGTAACTATCGTCTTGAGTCCAACCCGGTAAGACACGACTTATCGCCACTGGCAGCAGCCACTGGTAACAGGATTAGCAGAGCGA
GGTATGTAGGCGGTGCTACAGAGTTCTTGAAGTGGTGGCCTAACTACGGCTACACTAGAAGaACAGTATTTGGTATCTGCGCTCTGCTGAAGCCAGT
TACCTTCGGAAAAAGAGTTGGTAGCTCTTGATCCGGCAAACAAACCACCGCTGGTAGCGGTGGTTTTTTTGTTTGCAAGCAGCAGATTACGCGCAG
AAAAAAAGGATCTCAAGAAGATCCTTTGATCTTTTCTACGGGGTCTGACGCTCAGTGGAACGAAAACTCACGTTAAGGGATTTTGGTCATGAGATTAT
CAAAAAGGATCTTCACCTAGATCCTTTTAAATTAAAAATGAAGTTTTAAATCAATCTAAAGTATATATGAGTAAACTTGGTCTGACAGTTACCAATGCTTA
ATCAGTGAGGCACCTATCTCAGCGATCTGTCTATTTCGTTCATCCATAGTTGCCTGACTCCCCGTCGTGTAGATAACTACGATACGGGAGGGCTTACC
ATCTGGCCCCAGTGCTGCAATGATACCGCGAGACCCACGCTCACCGGCTCCAGATTTATCAGCAATAAACCAGCCAGCCGGAAGGGCCGAGCGCA
GAAGTGGTCCTGCAACTTTATCCGCCTCCATCCAGTCTATTAATTGTTGCCGGGAAGCTAGAGTAAGTAGTTCGCCAGTTAATAGTTTGCGCAACGTT
GTTGCCATTGCTACAGGCATCGTGGTGTCACGCTCGTCGTTTGGTATGGCTTCATTCAGCTCCGGTTCCCAACGATCAAGGCGAGTTACATGATCCC
CCATGTTGTGCAAAAAAGCGGTTAGCTCCTTCGGTCCTCCGATCGTTGTCAGAAGTAAGTTGGCCGCAGTGTTATCACTCATGGTTATGGCAGCACT
GCATAATTCTCTTACTGTCATGCCATCCGTAAGATGCTTTTCTGTGACTGGTGAGTACTCAACCAAGTCATTCTGAGAATAGTGTATGCGGCGACCGA
GTTGCTCTTGCCCGGCGTCAATACGGGATAATACCGCGCCACATAGCAGAACTTTAAAAGTGCTCATCATTGGAAAACGTTCTTCGGGGCGAAAACT
CTCAAGGATCTTACCGCTGTTGAGATCCAGTTCGATGTAACCCACTCGTGCACCCAACTGATCTTCAGCATCTTTTACTTTCACCAGCGTTTCTGGGT
GAGCAAAAACAGGAAGGCAAAATGCCGCAAAAAAGGGAATAAGGGCGACACGGAAATGTTGAATACTCATACTCTTCCTTTTTCAATATTATTGAAGC
ATTTATCAGGGTTATTGTCTCATGAGCGGATACATATTTGAATGTATTTAGAAAAATAAACAAATAGGGGTTCCGCGCACATTTCCCCGAAAAGTGCCAC
CTG

CAG promoter | HSPB3 export signal | *Sth*Csm direct repeat sequence

| | |
|---|---|
| Plasmid | pCX460 |
| Description | CRISPR array plasmid for *MAP1B* (Spacers 25-48) |

GgTCGACATTGATTATTGACTAGTTATTAATAGTAATCAATTACGGGGTCATTAGTTCATAGCCCATATATGGAGTTCCGCGTTACATAACTTACGGTAAAT
GGCCCGCCTGGCTGACCGCCCAACGACCCCCGCCCATTGACGTCAATAATGACGTATGTTCCCATAGTAACGCCAATAGGGACTTTCCATTGACGT
CAATGGGTGGAgTATTTACGGTAAACTGCCCACTTGGCAGTACATCAAGTGTATCATATGCCAAGTACGCCCCCTATTGACGTCAATGACGGTAAATG
GCCCGCCTGGCATTATGCCCAGTACATGACCTTATGGGACTTTCCTACTTGGCAGTACATCTACGTATTAGTCATCGCTATTACCATGGTCGAGGTGA
GCCCCACGTTCTGCTTCACTCTCCCCATCTCCCCCCCCcTCCCCACCCCCAATTTTGTATTTATTTATTTTTTAATTATTTTGTGCAGCGATGGGGGCG
GGGGGGGGGGGGGCGCGCGCCAGGCGGGGCGGGGCGGGGCGAGGGGCGGGGCGGGGCGAGGCGGAGAGGTGCGGCGGCAGCCAATCAGA
GCGGCGCGCTCCGAAAGTTTCCTTTTATGGCGAGGCGGCGGCGGCGGCGGCCCTATAAAAAGCGAAGCGCGCGGCGGGCGGGAGTCGCTGCGc
gcTGCCTTCGCCCCGTGCCCCGCTCCGCcGCCGCCTCGCGCCGCCCGCCCCGGCTCTGACTGACCGCGTTACTCCCACAGGTGAGCGGGCGGG
ACGGCCCTTCTCCTCCGGGCTGTAATTAGCGCTTGGTTTAATGACGGCTtGTTTCTTTTCTGTGGCTGCGTGAAAGCCTTgAgGGGCTCCGGGAGGG
CCCTTTGTGCGGGGGGAGCGGCTCGGGGGGTGCGTGCGTGTGTGTGTGCGTGGGGAGCGCCGCGTGCGGCtCCGCGCTGCCCGGCGGCTGTG
AGCGCTGCGGGCGCGGCGCGGGGCTTTGTGCGCTCCGCaGTGTGCGCGAGGGGAGCGCGGCCGGGGGCGGTGCCCCGCGGTGCGGGGGGG
gCTGCGAGGGGAACAAAGGCTGCGTGCGGGGTGTGTGCGTGGGGGGGTGAGCAGGGGGTGTGGGCGCGtCGGTCGGGCTGcAACCCCCCCTG
CACCCCCCTCCCCGAGTTGCTGAGCACGGCCCGGCTTCGGGTGCGGGGCTCCGTaCGGGGCGTGGCGCGGGGCTCGCCGTGCCGGGCGGGG
GGTGGCGGCAGGTGGGGGTGCCGGGCGGGGCGGGGCCGCCTCGGGCCGGGGAGGGCTCGGGGGAGGGGCGCGGCGGCCCCcGGAGCGCC
GGCGGCTGTCGAGGCGCGGCGAGCCGCAGCCATTGCCTTTTATGGTAATCGTGCGAGAGGGCGCAGGGACTTCCTTTGTCCCAAATCTGtGCGGA
GCCGAAATCTGGGAGGCGCCGCCGCACCCCCTCTAGCGGGCGCGGGgCGAAGCGGTGCGGCGCCGGCAGGAAGGAAATGGGCGGGGAGGGC
CTTCGTGCGTCGCCGCGCCGCCGTCCCCTTCTCCcTCTCCAGCCTCGGGGCTGtCCGCgGGGGGACGGCTGCCTTCGGGGGGGACGGGGCAGG
GCGGGGTTCGGCTTCTGGCGTGTGACCGGCGGCTCTAGAGCCTCTGCTAACCATGTTCATGCCTTCTTCTTTTTCCTACAGCTCCTGGGCAACGTG
CTGGTTaTTGTGCTGTCTCATCATTTTGGCAAAGAATTCgccaccgtggactcagcggcagagacgccaccccgagaaggcaaatcccactttcagatcctgctggacgtggtccagttcct
ccctgaagacatcatcattcagaccttcgaaggctggctgctgataaaagcacaacacggaaccagaatggatgagcacggttttatctcaagaagcttcacccgacagtacaaactaccagatggtgtggaaatca
aagatttgtctgcagtcctctgtcatgatggaattttggtggtggaagtaaaggatccagttgggactaagGAGCTCacctacgttgatgtatctctcagatataaacctaattacctcgagaggggacggaaacTCT
CCCCCACTCTCAAGTAGCACTTTTATGTTgatataaacctaattacctcgagaggggacggaaacAGAAGTATGTAGGATACCACTGGGTGATGTACgatataaacctaatta
cctcgagaggggacggaaacGAACCGTAGGAGTTGAGAATTCATTTGTTTTCgatataaacctaattacctcgagaggggacggaaacGAAAATCCCAAAGCAAATTTGTGC
TAGGGTTCgatataaacctaattacctcgagaggggacggaaacTAATCATGATCTGGCTTCCCAGGTTCACAGTTgatataaacctaattacctcgagaggggacggaaacACGGA
GAAGTTTGCCTCATCTGAACACTGGGTgatataaacctaattacctcgagaggggacggaaacTCATTGTTCTCTACTCCGTAGATCTTAGAGTCgatataaacctaattacct
cgagaggggacggaaacGACTTATCAGACAAAAATCAACTAAAATGTTAgatataaacctaattacctcgagaggggacggaaacTAGGTCCTCAGTGAAAGGACCCTGAA
GAAGCAgatataaacctaattacctcgagaggggacggaaacTTTCCAAGGACTTGAAAGAAATGGGGTAATAAgatataaacctaattacctcgagaggggacggaaacATATTAAT
TTTCTTTAAAAAATTAAACATTTGgatataaacctaattacctcgagaggggacggaaacACTCAATGAAATTCATTGGCGTCACAATGACTgatataaacctaattacctcgagag
gggacggaaacCCATTAAATAATTATCTTGAGTATCTTAGGTTgatataaacctaattacctcgagaggggacggaaacACATTTAATTGTGAAATTGGAAAATAACTTGCgat
ataaacctaattacctcgagaggggacggaaacTTGGCAAGAAGGCTAAAAATCTGGTTTTCTTCgatataaacctaattacctcgagaggggacggaaacAACTGAAGACTGGCT
ATAATTCTCCTACAATGgatataaacctaattacctcgagaggggacggaaacTTTACATATTTTTCCCATTAAATCCCATAGCAgatataaacctaattacctcgagaggggacggaa
acTGAGAACAACGGATGGAGAAAGGTACTGATAGgatataaacctaattacctcgagaggggacggaaacTGGAAGGACAGGAGCAACCTGTAATGCGAATAgatataa
acctaattacctcgagaggggacggaaacGGCTGTTACTAAACTAATCGTGTATTTATGGAgatataaacctaattacctcgagaggggacggaaacTGAACAAAGCTCCTAGAAG
TCTGTGTCGATCTgatataaacctaattacctcgagaggggacggaaacCATTTAAACAGAGTGTAAATTCCTTCCACTCAgatataaacctaattacctcgagaggggacggaaacC
TCTGTAGAATTTTGTTTAAGAATACTACCAAgatataaacctaattacctcgagaggggacggaaacCTCAGGGTTTTGAGAAAGGGGACAGAATTGGTgatataaacctaa
ttacctcgagaggggacggaaacAGGTTAACACTGCCCTTGAAGCTAGCAGATCTTTTTCCCTCTGCCAAAAATTATGGGGACATCATGAAGCCCCTTGAGCAT
CTGACTTCTGGCTAATAAAGGAAATTTATTTTCATTGCAATAGTGTGTTGGAATTTTTTGTGTCTCTCACTCGGAAGGACATATGGGAGGGCAAATCAT
TTAAAACATCAGAATGAGTATTTGGTTTAGAGTTTGGCAACATATGCCCATATGCTGGCTGCCATGAACAAAGGTTGGCTATAAAGAGGTCATCAGTAT
ATGAAACAGCCCCCTGCTGTCCATTCCTTATTCCATAGAAAAGCCTTGACTTGAGGTTAGATTTTTTTTATATTTTGTTTTGTGTTATTTTTTTCTTTAACA
TCCCTAAAATTTTCCTTACATGTTTTACTAGCCAGATTTTTCCTCCTCTCCTGACTACTCCCAGTCATAGCTGTCCCTCTTCTCTTATGgAGATCCCTCG
ACCTGCAGCCCAAGCTTGGCGTAATCATGGTCATAGCTGTTTCCTGTGTGAAATTGTTATCCGCTCACAATTCCACACAACATACGAGCCGGAAGCAT
AAAGTGTAAAGCCTGGGGTGCCTAATGAGTGAGCTAACTCACATTAATTGCGTTGCGCTCACTGCCCGCTTTCCAGTCGGGAAACCTGTCGTGCCA
GCGGATCCGCATCTCAATTAGTCAGCAACCATAGTCCCGCCCCTAACTCCGCCCATCCCGCCCCTAACTCCGCCCAGTTCCGCCCATTCTCCGCCC
CATGGCTGACTAATTTTTTTTATTTATGCAGAGGCCGAGGCCGCCTCGGCCTCTGAGCTATTCCAGAAGTAGTGAGGAGGCTTTTTTGGAGGCCTAG
GCTTTTGCAAAAAGCTAACTTGTTTATTGCAGCTTATAATGGTTACAAATAAAGCAATAGCATCACAAATTTCACAAATAAAGCATTTTTTTCACTGCATT
CTAGTTGTGGTTTGTCCAAACTCATCAATGTATCTTATCATGTCTGGATCCGCTGCATTAATGAATCGGCCAACGCGCGGGGAGAGGCGGTTTGCGTA
TTGGGCGCTCTTCCGCTTCCTCGCTCACTGACTCGCTGCGCTCGGTCGTTCGGCTGCGGCGAGCGGTATCAGCTCACTCAAAGGCGGTAATACGG
TTATCCACAGAATCAGGGGATAACGCAGGAAAGAACATGTGAGCAAAAGGCCAGCAAAAGGCCAGGAACCGTAAAAAGGCCGCGTTGCTGGCGTT
TTTCCATAGGCTCCGCCCCCCTGACGAGCATCACAAAAATCGACGCTCAAGTCAGAGGTGGCGAAACCCGACAGGACTATAAAGATACCAGGCGTT
TCCCCCTGGAAGCTCCCTCGTGCGCTCTCCTGTTCCGACCCTGCCGCTTACCGGATACCTGTCCGCCTTTCTCCCTTCGGGAAGCGTGGCGCTTT
CTCAtaGCTCACGCTGTAGGTATCTCAGTTCGGTGTAGGTCGTTCGCTCCAAGCTGGGCTGTGTGCACGAACCCCCCGTTCAGCCCGACCGCTGCG
CCTTATCCGGTAACTATCGTCTTGAGTCCAACCCGGTAAGACACGACTTATCGCCACTGGCAGCAGCCACTGGTAACAGGATTAGCAGAGCGAGGTA
TGTAGGCGGTGCTACAGAGTTCTTGAAGTGGTGGCCTAACTACGGCTACACTAGAAGaACAGTATTTGGTATCTGCGCTCTGCTGAAGCCAGTTACC
TTCGGAAAAAGAGTTGGTAGCTCTTGATCCGGCAAACAAACCACCGCTGGTAGCGGTGGTTTTTTTGTTTGCAAGCAGCAGATTACGCGCAGAAAA
AAAGGATCTCAAGAAGATCCTTTGATCTTTTCTACGGGGTCTGACGCTCAGTGGAACGAAAACTCACGTTAAGGGATTTTGGTCATGAGATTATCAAA
AAGGATCTTCACCTAGATCCTTTTAAATTAAAAATGAAGTTTTAAATCAATCTAAAGTATATATGAGTAAACTTGGTCTGACAGTTACCAATGCTTAATCA
GTGAGGCACCTATCTCAGCGATCTGTCTATTTCGTTCATCCATAGTTGCCTGACTCCCCGTCGTGTAGATAACTACGATACGGGAGGGCTTACCATCT
GGCCCCAGTGCTGCAATGATACCGCGAGACCCACGCTCACCGGCTCCAGATTTATCAGCAATAAACCAGCCAGCCGGAAGGGCCGAGCGCAGAAG
TGGTCCTGCAACTTTATCCGCCTCCATCCAGTCTATTAATTGTTGCCGGGAAGCTAGAGTAAGTAGTTCGCCAGTTAATAGTTTGCGCAACGTTGTTG
CCATTGCTACAGGCATCGTGGTGTCACGCTCGTCGTTTGGTATGGCTTCATTCAGCTCCGGTTCCCAACGATCAAGGCGAGTTACATGATCCCCCAT
GTTGTGCAAAAAAGCGGTTAGCTCCTTCGGTCCTCCGATCGTTGTCAGAAGTAAGTTGGCCGCAGTGTTATCACTCATGGTTATGGCAGCACTGCAT
AATTCTCTTACTGTCATGCCATCCGTAAGATGCTTTTCTGTGACTGGTGAGTACTCAACCAAGTCATTCTGAGAATAGTGTATGCGGCGACCGAGTTG
CTCTTGCCCGGCGTCAATACGGGATAATACCGCGCCACATAGCAGAACTTTAAAAGTGCTCATCATTGGAAAACGTTCTTCGGGGCGAAAACTCTCA
AGGATCTTACCGCTGTTGAGATCCAGTTCGATGTAACCCACTCGTGCACCCAACTGATCTTCAGCATCTTTTACTTTCACCAGCGTTTCTGGGTGAGC
AAAAACAGGAAGGCAAAATGCCGCAAAAAAGGGAATAAGGGCGACACGGAAATGTTGAATACTCATACTCTTCCTTTTTCAATATTATTGAAGCATTTA
TCAGGGTTATTGTCTCATGAGCGGATACATATTTGAATGTATTTAGAAAAATAAACAAATAGGGGTTCCGCGCACATTTCCCCGAAAAGTGCCACCTG

CAG promoter — HSPB3 export signal — *Sth*Csm direct repeat sequence

## Supplementary Table 3. smFISH probe sequences for *NOTCH2*, *MAP1B* and SthCsm pre-crRNA.

| FISH probe number | *NOTCH2* FISH probe Sequence |
|---|---|
| 1 | GGGATGTATTGAAGGAGGAT GTTACAAACCAATCATTTACATAACAGCAT A GGGATGTATTGAAGGAGGAT |
| 2 | GGGATGTATTGAAGGAGGAT TTGGAAGGCACCTTGTCCCTGAGCAACCAT A GGGATGTATTGAAGGAGGAT |
| 3 | GGGATGTATTGAAGGAGGAT CAGTTTTGTGATGTTTCCTATTTGACAGAT A GGGATGTATTGAAGGAGGAT |
| 4 | GGGATGTATTGAAGGAGGAT ACAACACCACTGAGCTTGGCAAACCTTTTT A GGGATGTATTGAAGGAGGAT |
| 5 | GGGATGTATTGAAGGAGGAT ACTACCTTTAGAATGAAACGGAAAAGTAAA A GGGATGTATTGAAGGAGGAT |
| 6 | GGGATGTATTGAAGGAGGAT TGCGGTCATTTTATTGGTTACTGGGTTTCT A GGGATGTATTGAAGGAGGAT |
| 7 | GGGATGTATTGAAGGAGGAT ATAGTACCACTTATTAGTGGGGGCCTCTGG A GGGATGTATTGAAGGAGGAT |
| 8 | GGGATGTATTGAAGGAGGAT GGTTAGGGCCAAAATCCCTAAACCACCTCT A GGGATGTATTGAAGGAGGAT |
| 9 | GGGATGTATTGAAGGAGGAT AATTGGCAAATTCAATAAGAGGATGCAATG A GGGATGTATTGAAGGAGGAT |
| 10 | GGGATGTATTGAAGGAGGAT TTCTCAGACTCTCTTTCCCTCATACCTTTC A GGGATGTATTGAAGGAGGAT |
| 11 | GGGATGTATTGAAGGAGGAT AACAAACAAAAAAACCTATCCCCAAAGGCA A GGGATGTATTGAAGGAGGAT |
| 12 | GGGATGTATTGAAGGAGGAT TTCAGAACCCAAATGAGAAAGTGATTATCC A GGGATGTATTGAAGGAGGAT |
| 13 | GGGATGTATTGAAGGAGGAT CTGTAATGACAGCTTTCACATTCCCTTTCT A GGGATGTATTGAAGGAGGAT |
| 14 | GGGATGTATTGAAGGAGGAT AACAAGCAATTTGGTCTGACATTGTGCTTA A GGGATGTATTGAAGGAGGAT |
| 15 | GGGATGTATTGAAGGAGGAT GCTGATACTTTCAGAAGTGGGGCTGGAGCA A GGGATGTATTGAAGGAGGAT |
| 16 | GGGATGTATTGAAGGAGGAT TCCATGCAGATAGGTTCTCCCCATCTGTGA A GGGATGTATTGAAGGAGGAT |
| 17 | GGGATGTATTGAAGGAGGAT TTGCACACTATGAAAGAGGCTGACAGAATG A GGGATGTATTGAAGGAGGAT |
| 18 | GGGATGTATTGAAGGAGGAT AGACACGCAGGGTTTCCATATGGGGCCACC A GGGATGTATTGAAGGAGGAT |
| 19 | GGGATGTATTGAAGGAGGAT GCCATAGAAGGAATGAGAACTGTCATTCAA A GGGATGTATTGAAGGAGGAT |
| 20 | GGGATGTATTGAAGGAGGAT AGGCAGAACTTATTTTGCAGGTGTCATGGG A GGGATGTATTGAAGGAGGAT |
| 21 | GGGATGTATTGAAGGAGGAT GGAGAAGAGGAAGAAAACTATTCTTTATCT A GGGATGTATTGAAGGAGGAT |
| 22 | GGGATGTATTGAAGGAGGAT AAATTGCCAAGAGCATGAATACAGAGAGTG A GGGATGTATTGAAGGAGGAT |
| 23 | GGGATGTATTGAAGGAGGAT CTATAGTTGTCCATTATCATCTAAAAGGTG A GGGATGTATTGAAGGAGGAT |
| 24 | GGGATGTATTGAAGGAGGAT AGTAACACGGACCACACGGTGTGAAAGAAA A GGGATGTATTGAAGGAGGAT |
| 25 | GGGATGTATTGAAGGAGGAT GAGACTGATCATCTGACAAACGGAAGACAA A GGGATGTATTGAAGGAGGAT |
| 26 | GGGATGTATTGAAGGAGGAT AATGGCAAGGGTACAACATTTGAGACAGTG A GGGATGTATTGAAGGAGGAT |
| 27 | GGGATGTATTGAAGGAGGAT TTGCACGAGTTAACTTGTCTCTTGGTCATT A GGGATGTATTGAAGGAGGAT |
| 28 | GGGATGTATTGAAGGAGGAT GTCATAGTAACTAGACTATCAAGGATAAAA A GGGATGTATTGAAGGAGGAT |
| 29 | GGGATGTATTGAAGGAGGAT GGTGCAGCCTCTTGAAGGGATCTCCTGCCC A GGGATGTATTGAAGGAGGAT |
| 30 | GGGATGTATTGAAGGAGGAT AAAATTTGTTGAAGAAAAGTATTCTTCTCC A GGGATGTATTGAAGGAGGAT |
| 31 | GGGATGTATTGAAGGAGGAT GTATGTCCATTTTCCAAAGAAACAACATAC A GGGATGTATTGAAGGAGGAT |
| 32 | GGGATGTATTGAAGGAGGAT GATACCGGGAAGACAGGAGGGGAAAGGAGA A GGGATGTATTGAAGGAGGAT |
| 33 | GGGATGTATTGAAGGAGGAT TATAACATGCTGGTAGGGCTACAATCACGG A GGGATGTATTGAAGGAGGAT |
| 34 | GGGATGTATTGAAGGAGGAT CCAAAAGCACCAAATGAAGACAAAAGAATG A GGGATGTATTGAAGGAGGAT |
| 35 | GGGATGTATTGAAGGAGGAT GAGATGCGTAGGTCAATTCAGGCAGAATTC A GGGATGTATTGAAGGAGGAT |
| 36 | GGGATGTATTGAAGGAGGAT CATAATTCCCAACAGGACGCTAGTGTAGAA A GGGATGTATTGAAGGAGGAT |
| 37 | GGGATGTATTGAAGGAGGAT ATGCAGTCCAAGCTGCAAGAATGTCTGGGC A GGGATGTATTGAAGGAGGAT |
| 38 | GGGATGTATTGAAGGAGGAT TAGATTAGAATAATCAATAAGCCTTGCAGA A GGGATGTATTGAAGGAGGAT |
| 39 | GGGATGTATTGAAGGAGGAT CTACACTGGAGGTGGACTCTCTCACGCATA A GGGATGTATTGAAGGAGGAT |

| | |
|---|---|
| 40 | GGGATGTATTGAAGGAGGAT GGCTGCTATATAGTTCCTCCTTTTCCAACC A GGGATGTATTGAAGGAGGAT |
| FISH probe number | *MAP1B* FISH probe Sequence |
| 1 | GGGATGTATTGAAGGAGGAT A AAGACCACTTCTTTCCAGTGCTGGAAAGAG A GGGATGTATTGAAGGAGGAT |
| 2 | GGGATGTATTGAAGGAGGAT A AGTGTAGGAAAAGCGTGTCTCTGAACTGCC A GGGATGTATTGAAGGAGGAT |
| 3 | GGGATGTATTGAAGGAGGAT A TCCAAACAAGTGCAGAGAAGCTGCCTATTT A GGGATGTATTGAAGGAGGAT |
| 4 | GGGATGTATTGAAGGAGGAT A AATCAACTTCATGAAGTCACAGATTTTTTA A GGGATGTATTGAAGGAGGAT |
| 5 | GGGATGTATTGAAGGAGGAT A TAGTTTTAATACCAGGTGAATAACCTAATT A GGGATGTATTGAAGGAGGAT |
| 6 | GGGATGTATTGAAGGAGGAT A TCAAAGAAATGCTCATCCCTAGGCTGCTTT A GGGATGTATTGAAGGAGGAT |
| 7 | GGGATGTATTGAAGGAGGAT A AAAGCTTACAGTTCCTTTCAAATGGAAACA A GGGATGTATTGAAGGAGGAT |
| 8 | GGGATGTATTGAAGGAGGAT A CTAACTCACAATATCATTACTGAGCAGGAA A GGGATGTATTGAAGGAGGAT |
| 9 | GGGATGTATTGAAGGAGGAT A ATTCAGAATTGTAGAGCAGCTCCTAAGAGC A GGGATGTATTGAAGGAGGAT |
| 10 | GGGATGTATTGAAGGAGGAT A AATGAAAGGTGGTAAGATTTCCATTAGCAA A GGGATGTATTGAAGGAGGAT |
| 11 | GGGATGTATTGAAGGAGGAT A ATTAAATGAGTATTGGAAAGCTCCTAGACT A GGGATGTATTGAAGGAGGAT |
| 12 | GGGATGTATTGAAGGAGGAT A TCCAGTCCCACCACAAGACTGAGAAAGCAT A GGGATGTATTGAAGGAGGAT |
| 13 | GGGATGTATTGAAGGAGGAT A TGAAAATGGAGGATCATCGCTAACATTTAG A GGGATGTATTGAAGGAGGAT |
| 14 | GGGATGTATTGAAGGAGGAT A AGGTCATGATCATGAGTGCTATCTCGATAG A GGGATGTATTGAAGGAGGAT |
| 15 | GGGATGTATTGAAGGAGGAT A GCTCTCACCATGAATGTACATGGTACATTG A GGGATGTATTGAAGGAGGAT |
| 16 | GGGATGTATTGAAGGAGGAT A AAAATTAGGTTAGTCACACAGGCAGGAAAG A GGGATGTATTGAAGGAGGAT |
| 17 | GGGATGTATTGAAGGAGGAT A GGTTTTCTTGGTATACTCAGGCTCTGTGGT A GGGATGTATTGAAGGAGGAT |
| 18 | GGGATGTATTGAAGGAGGAT A CTGTCAAAGAAAAATCAACCAGAGGTCTGG A GGGATGTATTGAAGGAGGAT |
| 19 | GGGATGTATTGAAGGAGGAT A CGCATTATGTAGACCAATTCAACTTAAGAG A GGGATGTATTGAAGGAGGAT |
| 20 | GGGATGTATTGAAGGAGGAT A TCCATCATGTGTTAATTTCTTGTACCCTTA A GGGATGTATTGAAGGAGGAT |
| 21 | GGGATGTATTGAAGGAGGAT A AAAATGTAATTCACAGCATTAAGTAGACTG A GGGATGTATTGAAGGAGGAT |
| 22 | GGGATGTATTGAAGGAGGAT A TTAAACCCTCATCATAGTTAGCAGTGCAAA A GGGATGTATTGAAGGAGGAT |
| 23 | GGGATGTATTGAAGGAGGAT A AGGAAATGTAATGTGACAGAGCACAGCACA A GGGATGTATTGAAGGAGGAT |
| 24 | GGGATGTATTGAAGGAGGAT A CATTGATCTGCAACTTGAAACTTGATGTCA A GGGATGTATTGAAGGAGGAT |
| 25 | GGGATGTATTGAAGGAGGAT A TGACAATGATGGAAATTTTCTCTAAACTCT A GGGATGTATTGAAGGAGGAT |
| 26 | GGGATGTATTGAAGGAGGAT A TGTCAAGGCCCTGAAAAGTAGCCTACCATG A GGGATGTATTGAAGGAGGAT |
| 27 | GGGATGTATTGAAGGAGGAT A AATCAAGGGGTGGGAGGTATCATTATTCCT A GGGATGTATTGAAGGAGGAT |
| 28 | GGGATGTATTGAAGGAGGAT A ATCAGACTTCAATCTTGCCAGAACTTCAAA A GGGATGTATTGAAGGAGGAT |
| 29 | GGGATGTATTGAAGGAGGAT A GCCATTTCAGTCTTGAACTTGCAGTAGTAA A GGGATGTATTGAAGGAGGAT |
| 30 | GGGATGTATTGAAGGAGGAT A GCCACAGGTGCACTGAAGTGGGATTCAACA A GGGATGTATTGAAGGAGGAT |
| 31 | GGGATGTATTGAAGGAGGAT A TGTTTTATAAACTTGCTGTCTCACATTAAA A GGGATGTATTGAAGGAGGAT |
| 32 | GGGATGTATTGAAGGAGGAT A TTCCTAAGGCAAATTGTCTACCATATGTAC A GGGATGTATTGAAGGAGGAT |
| 33 | GGGATGTATTGAAGGAGGAT A GGACTTGGGTGTTGCTGCATGCAGGTTAAT A GGGATGTATTGAAGGAGGAT |
| 34 | GGGATGTATTGAAGGAGGAT A AGCATGCCTTAGATCAATCTACTTGTGTGT A GGGATGTATTGAAGGAGGAT |
| 35 | GGGATGTATTGAAGGAGGAT A ATGGCCAGTCTACACTCACTACATTATGTC A GGGATGTATTGAAGGAGGAT |
| 36 | GGGATGTATTGAAGGAGGAT A GATGTGAAAATATCACATGCCCTCATAACA A GGGATGTATTGAAGGAGGAT |
| 37 | GGGATGTATTGAAGGAGGAT A TAAAGAATGCCTACTGCCTGAGAAAATGCA A GGGATGTATTGAAGGAGGAT |
| 38 | GGGATGTATTGAAGGAGGAT A GTTGGCTAGTGCATGGTTTCCAAATGTTCA A GGGATGTATTGAAGGAGGAT |
| 39 | GGGATGTATTGAAGGAGGAT A TTGAACCCTATTGTGGAAATACTGTTATTT A GGGATGTATTGAAGGAGGAT |
| 40 | GGGATGTATTGAAGGAGGAT A CCTTGGTTTTTACAGTTCAATCTTGCATGC A GGGATGTATTGAAGGAGGAT |
| 41 | GGGATGTATTGAAGGAGGAT A TGCTGCTTGCTAAAACCATGATGTTGAGAT A GGGATGTATTGAAGGAGGAT |

| | |
|---|---|
| 42 | GGGATGTATTGAAGGAGGAT A TCATCACTTCTGAGTCATGAGTTGGGATCA A GGGATGTATTGAAGGAGGAT |
| 43 | GGGATGTATTGAAGGAGGAT A TCATTCCCACTCACCACGTAGTAGGAAGAC A GGGATGTATTGAAGGAGGAT |
| 44 | GGGATGTATTGAAGGAGGAT A TTACTATTGCTGTGGTTAGGAATGTAGCAC A GGGATGTATTGAAGGAGGAT |
| SthCsm pre-crRNA FISH probe sequence | GGGATGTATTGAAGGAGGAT AA CCGTCCCCTCTCGAGGTAATTAGGTTTATATC AA GGGATGTATTGAAGGAGGAT |
| Readout probe sequence | ATTO590-ATCCTCCTTCAATACATCCC |

## Supplementary Table 4. qPCR primers.

| RNA abundance measurement | |
|---|---|
| Primer name | Sequence |
| NOTCH2_3'UTR_F1 | TATTTGGGAGGACACATTTATGTACCCAGAG |
| NOTCH2_3'UTR_R1 | GGTTACTGGGTTTCTCATACAAACAGATATAATATCACTT |
| NOTCH2_3'UTR_F2 | GCATCCTCTTATTGAATTTGCCAATTTGTAATTTTTGC |
| NOTCH2_3'UTR_R2 | GTCATCATACATTCAATGTATTGGTTAGGGCC |
| MAP1B_3'UTR_F1 | TATGGTATTATGTCTTATAATCCTGCATCACTTCTATCCT |
| MAP1B_3'UTR_R1 | CCTTTACATCAAATATCCTCAATGGAAGAGGGG |
| MAP1B_3'UTR_F2 | AAAACAGGAGCCATGGAGAAGTGG |
| MAP1B_3'UTR_R2 | AAGTGCTTCTCAGCTGGGCTCAAGA |
| NOTCH2_ORF_F1 | ACAGTTGTGTCTGCTCACCAGGAT |
| NOTCH2_ORF_R1 | GCGGAAACCATTCACACCGTTGAT |
| NOTCH2_ORF_F2 | AAAAATGGGGCCAACCGAGAC |
| NOTCH2_ORF_R2 | TTCATCCAGAAGGCGCACAA |
| MAP1B_ORF_F1 | TGGGACACAAACCTGATTGA |
| MAP1B_ORF_R1 | AAACGTCACTTCGGTGATGA |
| MAP1B_ORF_F2 | CAAGAGAGTGCGGTCTTCCTAC |
| MAP1B_ORF_R2 | CTGAGTCATGAGTTGGGATCAG |
| GAPDH_F | CCAGAACATCATCCCTGCCTCTACTG |
| GAPDH_R | GGAAATGAGCTTGACAAAGTGGTCGTTG |

| RNA decay measurement | |
|---|---|
| Primer name | Sequence |
| NOTCH2_F | ACAGTTGTGTCTGCTCACCAGGAT |
| NOTCH2_R | GCGGAAACCATTCACACCGTTGAT |
| MAP1B_F | TGGGACACAAACCTGATTGA |
| MAP1B_R | AAACGTCACTTCGGTGATGA |
| 18S_F | CTTGGAGGGACAAGTGGCG |
| 18S_R | ACGCTGAGCCAGTCAGTGTA |
