# Supplementary material for: Single-molecule live-cell RNA imaging with CRISPR–Csm
Source: Nat Biotechnol. 2025 Feb 18;43(12):2023–30. doi: 10.1038/s41587-024-02540-5 (PMC12700784; doi:10.1038/s41587-024-02540-5)
Supplement: Supplementary file 2 — Reporting Summary [file 41587_2024_2540_MOESM2_ESM.pdf]

nature portfolio

Corresponding author(s): Jennifer A. Doudna

Last updated by author(s): Dec 9, 2024

Reporting Summary

Nature Portfolio wishes to improve the reproducibility of the work that we publish. This form provides structure for consistency and transparency in reporting. For further information on Nature Portfolio policies, see our Editorial Policies and the Editorial Policy Checklist.

Statistics

For all statistical analyses, confirm that the following items are present in the figure legend, table legend, main text, or Methods section.

n/a	Confirmed	
☐	☒	The exact sample size (*n*) for each experimental group/condition, given as a discrete number and unit of measurement
☒	☐	A statement on whether measurements were taken from distinct samples or whether the same sample was measured repeatedly
☐	☒	The statistical test(s) used AND whether they are one- or two-sided<br>*Only common tests should be described solely by name; describe more complex techniques in the Methods section.*
☒	☐	A description of all covariates tested
☒	☐	A description of any assumptions or corrections, such as tests of normality and adjustment for multiple comparisons
☐	☒	A full description of the statistical parameters including central tendency (e.g. means) or other basic estimates (e.g. regression coefficient) AND variation (e.g. standard deviation) or associated estimates of uncertainty (e.g. confidence intervals)
☐	☒	For null hypothesis testing, the test statistic (e.g. *F*, *t*, *r*) with confidence intervals, effect sizes, degrees of freedom and *P* value noted<br>*Give P values as exact values whenever suitable.*
☒	☐	For Bayesian analysis, information on the choice of priors and Markov chain Monte Carlo settings
☒	☐	For hierarchical and complex designs, identification of the appropriate level for tests and full reporting of outcomes
☒	☐	Estimates of effect sizes (e.g. Cohen's *d*, Pearson's *r*), indicating how they were calculated

Our web collection on statistics for biologists contains articles on many of the points above.

Software and code

Policy information about availability of computer code

Data collection	Zen Pro 2012, CFX96 Real-Time PCR Detection System (Bio-Rad), LI-COR Odyssey CLx, bioRender
Data analysis	FIJI(ImageJ V 2.14.0), ComDet (V 0.5.5), TrackMate (V 7.12.1), GraphPad Prism (V 10.2.2), KymoResliceWide (FIJI plugin V 0.6.0)

For manuscripts utilizing custom algorithms or software that are central to the research but not yet described in published literature, software must be made available to editors and reviewers. We strongly encourage code deposition in a community repository (e.g. GitHub). See the Nature Portfolio guidelines for submitting code & software for further information.

Data

Policy information about availability of data

All manuscripts must include a data availability statement. This statement should provide the following information, where applicable:

- Accession codes, unique identifiers, or web links for publicly available datasets
- A description of any restrictions on data availability
- For clinical datasets or third party data, please ensure that the statement adheres to our policy

Essential plasmids have been deposited at Addgene (plasmid ID: 229211- 229216). Unprocessed microscope image files have been deposited at figshare (doi 10.6084/m9.figshare.27997130).

Research involving human participants, their data, or biological material

Policy information about studies with human participants or human data. See also policy information about sex, gender (identity/presentation), and sexual orientation and race, ethnicity and racism.

Reporting on sex and gender	This study did not involve human research participants.
Reporting on race, ethnicity, or other socially relevant groupings	N.A.
Population characteristics	N.A.
Recruitment	N.A.
Ethics oversight	N.A.

Note that full information on the approval of the study protocol must also be provided in the manuscript.

Field-specific reporting

Please select the one below that is the best fit for your research. If you are not sure, read the appropriate sections before making your selection.

☒ Life sciences ☐ Behavioural & social sciences ☐ Ecological, evolutionary & environmental sciences

For a reference copy of the document with all sections, see nature.com/documents/nr-reporting-summary-flat.pdf

Life sciences study design

All studies must disclose on these points even when the disclosure is negative.

Sample size	No sample size calculation was performed in this study. All experiments were performed with three or more biological replicates. 31 cells were used for co-localization calculation and 275 cells have been used for for labeling efficiency calculation. RNA abundance, decay, protein level, and localization measurements ere performed with three or more biological replicates. This information is extensively described in the figure captions. Sample sizes were chosen based on the standard in the field and prior knowledge of experimental variation.
Data exclusions	No data were excluded.
Replication	All experiments were performed with three or more biological replicates. All attempts at replication were successful.
Randomization	Randomization was unnecessary for this study. Because our study relied solely on objective, quantitative measurements conducted under controlled conditions without the use of animal or human subjects. Randomization is not commonly utilized in these types of investigations.
Blinding	Blinding was unnecessary for this study. Because our study relied solely on objective, quantitative measurements conducted under controlled conditions. And all the experimental settings and conditions need to be clear to the authors.

Reporting for specific materials, systems and methods

We require information from authors about some types of materials, experimental systems and methods used in many studies. Here, indicate whether each material, system or method listed is relevant to your study. If you are not sure if a list item applies to your research, read the appropriate section before selecting a response.

Materials & experimental systems

n/a	Involved in the study	
☐	☒	Antibodies
☐	☒	Eukaryotic cell lines
☒	☐	Palaeontology and archaeology
☒	☐	Animals and other organisms
☒	☐	Clinical data
☒	☐	Dual use research of concern
☒	☐	Plants

Methods

n/a	Involved in the study	
☒	☐	ChIP-seq
☒	☐	Flow cytometry
☒	☐	MRI-based neuroimaging

Antibodies

Antibodies used	Ubiquityl-Histone H2A (Lys119) (D27C4) Rabbit mAb (Cell Signaling Technology), DCP1A antibody (Abcam, ab183709), NOTCH2 antibody (Cell Signaling, 5732S), MAP1B antibody (Thermo Fisher Scientific, PA5-82798), ACTB antibody (Proteintech, 60008-1-Ig), IRDye 680RD goat anti-mouse (LI-COR, 926-68070), IRDye 800CW goat anti-rabbit (LI-COR, 926-32211)
Validation	The primary antibodies listed above have been extensively used in the field and validated by the manufacturer. Please see citations and data on each of the product pages (listed below) demonstrating antibody validity for the purposes of Western blot and immunofluorescence.<br>Ubiquityl-Histone H2A : https://www.cellsignal.com/products/primary-antibodies/ubiquityl-histone-h2a-lys119-d27c4-xp-rabbit-mab/8240.<br>DCP1A: https://www.abcam.com/en-us/products/primary-antibodies/dcp1a-antibody-epr13822-ab183709.<br>NOTCH2: https://www.cellsignal.com/products/primary-antibodies/notch2-d76a6-xp-rabbit-mab/5732.<br>MAP1B : https://www.thermofisher.com/antibody/product/MAP1B-Antibody-Polyclonal/PA5-82798.<br>ACTB: https://www.ptglab.com/products/ACTB-Antibody-60008-1-Ig.htm .

Eukaryotic cell lines

Policy information about cell lines and Sex and Gender in Research

Cell line source(s)	HEK293T, U2OS, HeLa, IMR-90 and COS-7 cells were obtained from the UC Berkeley Cell Culture Facility
Authentication	All cell lines were authenticated by STR profiling and purchased commercially from the UC Berkeley Cell Culture Facility.
Mycoplasma contamination	All cell lines were confirmed to be mycoplasma-free (abm, PCR mycoplasma detection kit).
Commonly misidentified lines<br>(See ICLAC register)	No commonly misidentified cell lines were used in the study.

Plants

Seed stocks	This study did not involve plants.
Novel plant genotypes	N.A.
Authentication	N.A.
