# Supplementary figures and images for: Single-molecule live-cell RNA imaging with CRISPR–Csm
Source: Nat Biotechnol. 2025 Feb 18;43(12):2023–30. doi: 10.1038/s41587-024-02540-5 (PMC12700784; doi:10.1038/s41587-024-02540-5)

# Unprocessed Western blots

## Extended Data Fig. 7c

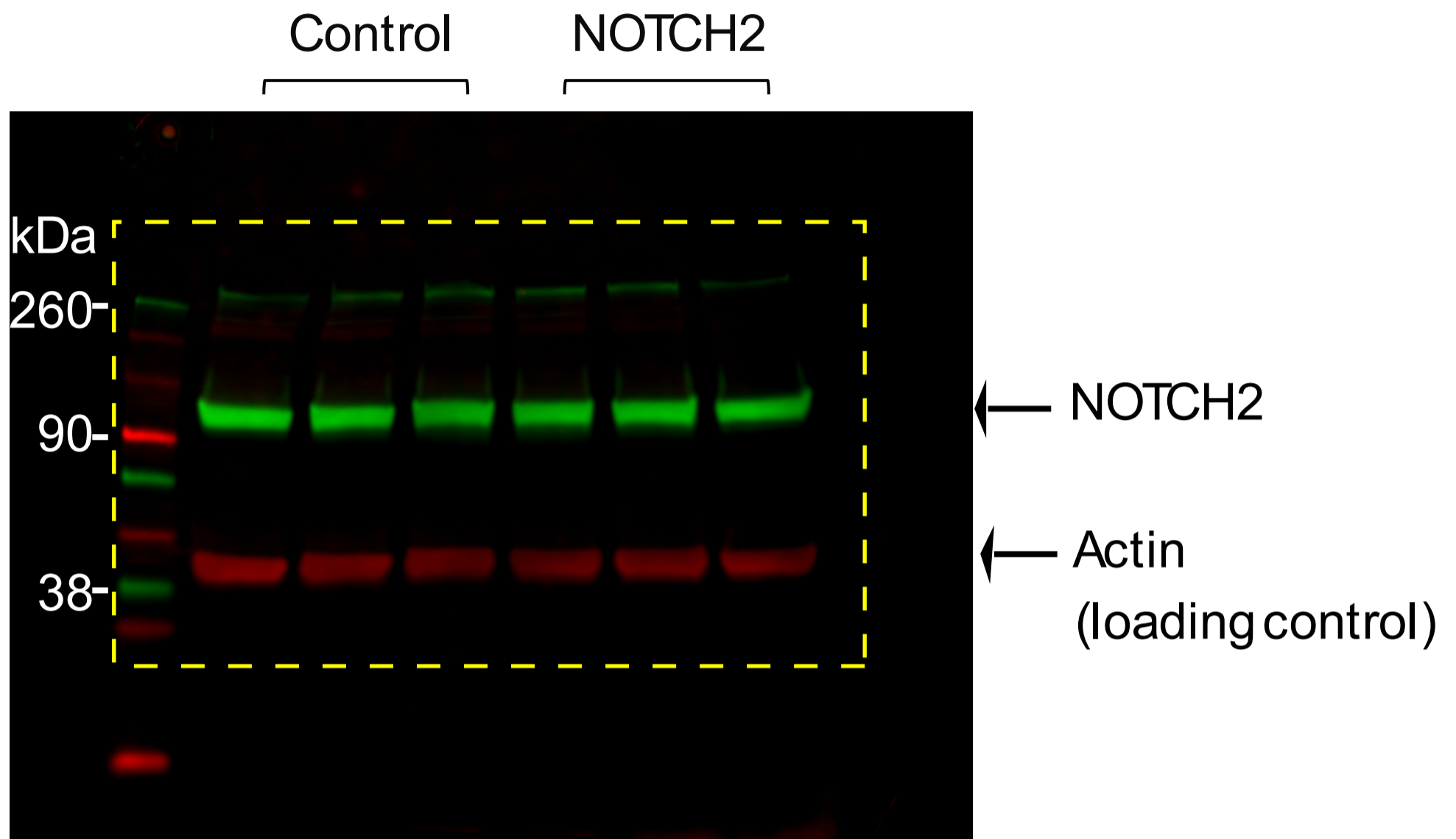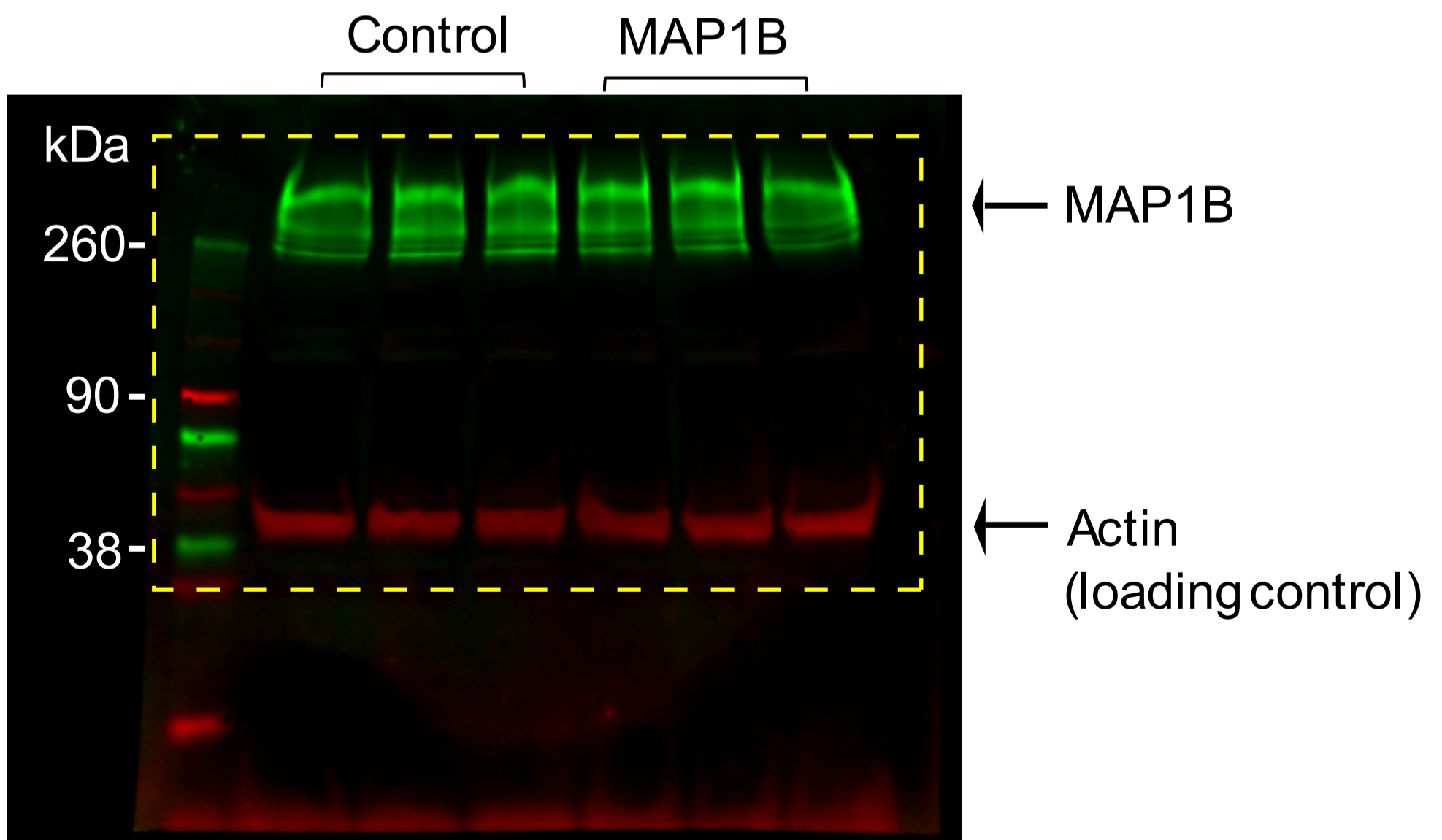

Supplement: Supplementary file 7 — Unprocessed western blots. [file 41587_2024_2540_MOESM7_ESM.pdf]
